# Abnormal behaviors and glial responses in an animal model of tau pathology

**DOI:** 10.1186/s13041-025-01252-4

**Published:** 2025-11-06

**Authors:** Yue Liu, Akira Sobue, Naruhiko Sahara, Madoka Isobe, Rinako Tanaka, Youyun Zhu, Wenjun Zhu, Tetsuo Matsuzaki, Koji Yamanaka, Kiyofumi Yamada, Hiroyuki Mizoguchi

**Affiliations:** 1 Department of Neuropsychopharmacology and Hospital Pharmacy, Nagoya University Graduate School of Medicine, Nagoya, Japan; 2 Department of Neuroscience & Pathobiology, Research Institute of Environmental Medicine, Nagoya University, Nagoya, Japan; 3 Medical Interactive Research and Academia-Industry Collaboration Center, Research Institute of Environmental Medicine, Nagoya University, Nagoya, Japan; 4 Division of Behavioral Neuropharmacology, Fujita Mind-Brain Research & Innovation Center for Drug Generation (Fujita Mind-BRIDGe), Fujita Health University, Toyoake, Japan; 5 Division of Behavioral Neuropharmacology, International Center for Brain Science (ICBS), Fujita Health University, Toyoake, Japan; 6 Advanced Neuroimaging Center, Institute for Quantum Medical Sciences, National Institutes for Quantum Science and Technology, Chiba, Japan; 7 Center for Integrated Human Brain Science, Brain Research Institute, Niigata University, Niigata, Japan; 8 Institute for Glyco-Core Research (iGCORE), Nagoya University, Nagoya, Japan; 9 Center for One Medicine Innovative Translational Research (COMIT), Nagoya University, Nagoya, Japan; 10 Laboratory of Health and Medical Science Innovation, Fujita Health University Graduate School of Medical Sciences, Toyoake, Japan

**Keywords:** Alzheimer’s disease, Tau, Glia, Neuroinflammation, Behavior

## Abstract

Tau hyperphosphorylation has been considered a major contributor to neurodegeneration in Alzheimer’s disease (AD) and frontotemporal dementia, and related tauopathies have gained prominence in the development of therapies for these conditions. Glial responses are key features of AD and frontotemporal dementia, and are associated with neuroinflammation. Numerous transgenic mouse models that recapitulate critical AD-like pathology and cognitive impairment have been developed to examine pathogenic mechanisms and evaluate therapeutic approaches targeting tau and glial reactivity. Glial reactivity and neuroinflammation coincide with tau hyperphosphorylation, which induces behavioral impairment; however, the specific correlation between glial cell activation and abnormal behavior remains unknown. In this study, we investigated changes in glial cell gene expressions related to abnormal behaviors in rTg4510 mice, which phenocopy the tau pathology, neuroinflammation, and neurodegeneration observed in human tauopathies. Both 4- and 6-month-old rTg4510 mice displayed significantly impaired nest-building behavior compared with control mice. Paired association learning was also impaired in 4-month-old rTg4510 mice. Moreover, rTg4510 mice of both age groups exhibited abnormal exploratory behavior, and these mice spent a longer time in the open arms of the plus-maze test than control mice. Using a magnetic-activated cell-sorting technique, we analyzed glial cell gene expressions related to neuroinflammation, phagocytosis, and amyloid synthesis in the prefrontal cortex of rTg4510 mice. Regression analysis of glial gene expressions and behavioral tests revealed that various glial reactivities were associated with behavioral abnormalities. Our findings suggest specific genetic characteristics of glial cells that may lead to abnormal behavior in rTg4510 mice.

**Supplementary Information:**

The online version contains supplementary material available at 10.1186/s13041-025-01252-4.

## Introduction

Neurodegenerative diseases (e.g., Alzheimer’s disease [AD] and frontotemporal dementia) are clinically characterized by disease-associated neuropathological processes as well as serious impairments in cognitive and sensorimotor function [1–3]. These processes are commonly associated with age-related protein aggregation. For instance, AD is marked by the presence of extracellular senile plaques, composed of amyloid-β (Aβ) peptides, and intracellular neurofibrillary tangles (NFTs), which consist of bundles of paired helical filaments of the microtubule-associated protein tau [1]. The molecular mechanisms linking the aggregation of these proteins and neurodegeneration remain to be elucidated.

Prominent abnormal behaviors in AD and frontotemporal dementia include progressive memory loss and cognitive decline. While cognitive deficits are common features of both conditions, many patients also exhibit behavioral and psychological symptoms of dementia (BPSD), including depression, anxiety, and reduced sociability [4, 5]. Deteriorations of executive functions and daily life activities are also early signs of AD, and constitute a significant source of distress for patients and caregivers [6, 7]. Behavioral tests in many mouse models of AD provide an effective means of measuring cognitive impairment, BPSD, and daily life activity. We also found that touchscreen-based tasks are useful to assess cognitive impairment by detecting AD-associated behavioral impairments with high sensitivity at an early stage [8].

Neuroinflammation is an important factor in the neurodegeneration process, and is a prominent aspect of AD and frontotemporal dementia [9]. Aβ aggregates and abnormal tau phosphorylation are common pathological phenomena in AD that can induce neuroinflammation, further accelerating protein aggregation and neurodegeneration. Recently, neuroinflammation has been found to be not only a result of neurodegeneration, but also a crucial player in the process of protein aggregation, and it is considered to contribute to behavioral changes [10, 11]. Thus, neuroinflammation can trigger neurodegeneration and suggests promising therapeutic avenues to limit neurodegenerative processes. Chronic neuroinflammation can be induced by activated microglia and astrocytes through the release of multiple neurotoxic factors in response to protein aggregates, ultimately leading to neurodegeneration [12]. Distinct glial cell responses against amyloid and tau pathologies have been identified in AD [13]. In AD mice model, it has become possible to isolate specific cell types and to observe changes in the expression levels of pro-inflammatory genes in glial cells; however, the correlations between gene expression changes in certain cell types and behavioral abnormalities are not fully understood.

In this study, we chose the rTg4510 mouse model to examine glial activation during the neurodegenerative process. rTg4510 mice express human tau containing the P301L mutation, which has been linked with frontotemporal dementia, and they mimic the features of human tauopathy, including tau hyperphosphorylation, neuronal loss, and memory impairment [14]. Thus rTg4510 mice have been used as an essential model for investigating tau-related dysfunction, neuroinflammation, and associated behavioral deficits [15]. Using the rTg4510 mouse model, we analyzed BPSD and cognition, and also the expression levels of candidate genes, including markers of inflammation and disease-associated microglia (DAM), in the glial cells of the frontal cortex. Furthermore, we evaluated the correlation between behavioral abnormalities and the expression levels of these genes to clarify the relationship between tau-induced neuroinflammation and behavioral impairment in tau pathology.

## Results

### rTg4510 mice, characterized by elevated levels of phosphorylated tau, display abnormal behavior as early as 4 months of age, with notable worsening by 6 months of age

**Fig. 1:**
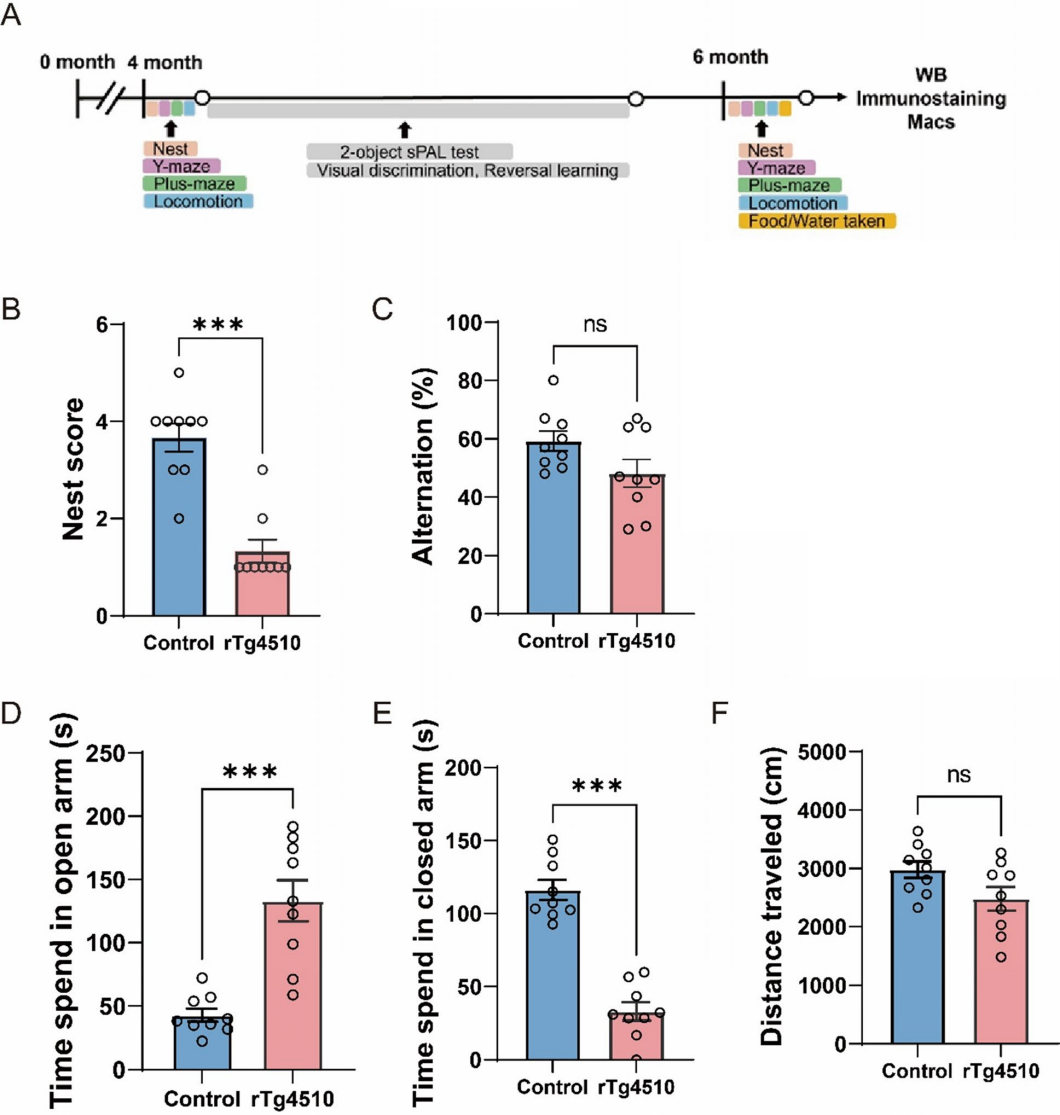
Four-month-old rTg4510 mice exhibited abnormal behavior. **A** Behavioral tests performed at different time points. **B** Nesting scores in 4-month-old mice. Data are presented as means ± SEM. ***P < 0.001. **C** Spontaneous alternation of 4-month-old mice in the Y-maze test. **D** Time spent by 4-month-old mice in open arms in the plus-maze test. Data are presented as means ± SEM. ***P < 0.001. **E,** Time spent by 4-month-old mice in closed arms in the plus-maze test. Data are presented as means ± SEM. ***P < 0.001. **F** Total distance traveled by 4-month-old mice in both open and closed arms in the plus-maze test. The control group is Camk2a-tTA mouse. Male (control: n = 4, rTg4510: n = 4) and female (control: n = 5, rTg4510: n = 5) mice

**Fig. 2:**
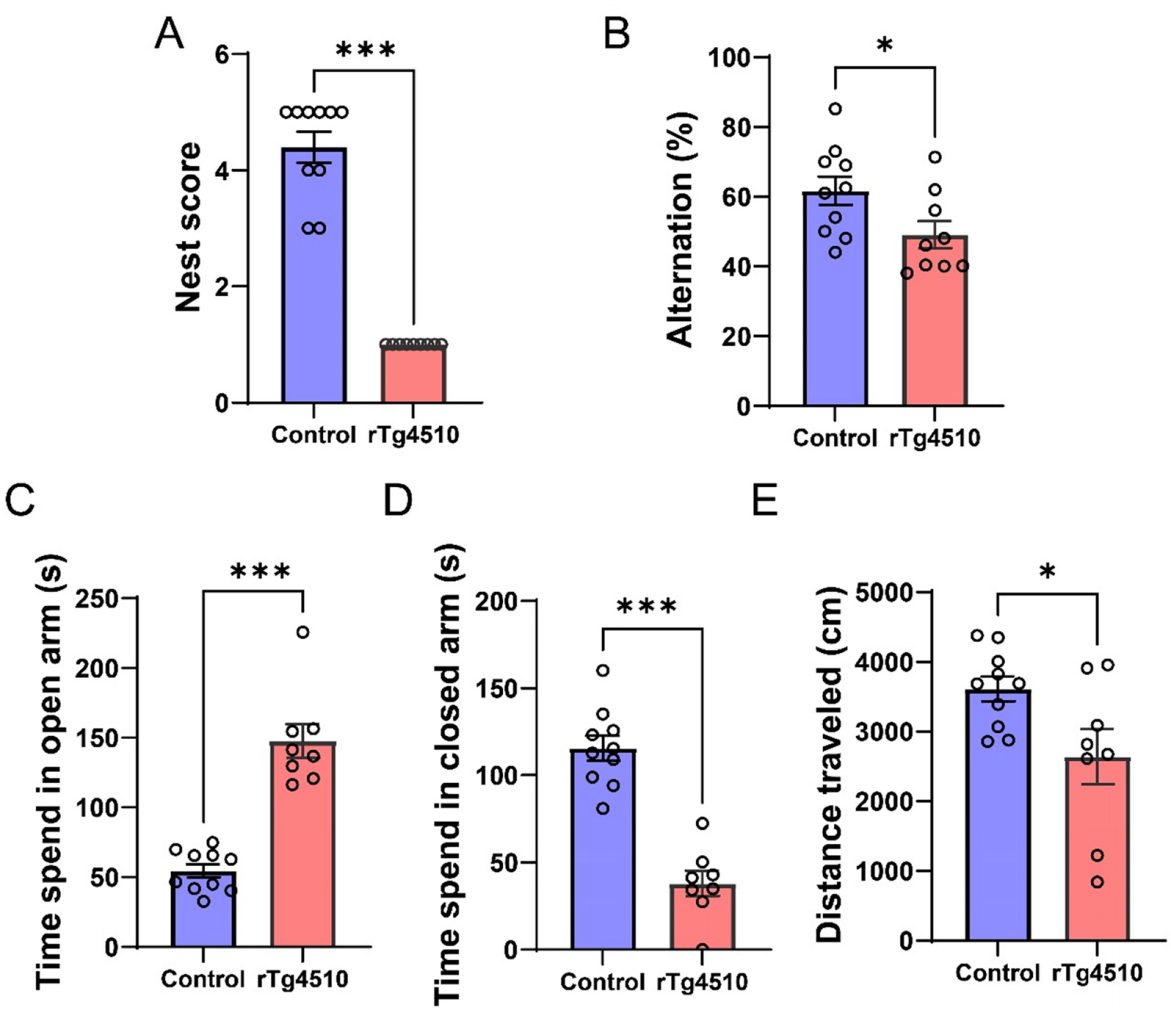
Abnormal behaviors exhibited by rTg4510 mice worsen as they reach 6 months of age. **A** Nesting scores in 6-month-old mice. Data are presented as means ± SEM. ***P < 0.001. **B** Spontaneous alternation of 6-month-old mice in the Y-maze test. Data are presented as means ± SEM. *P < 0.05. **C** Time spent by 6-month-old mice in open arms in the plus-maze test. Data are presented as means ± SEM. ***P < 0.001. **D** Time spent by 6-month-old mice in closed arms in the plus-maze test. Data are presented as means ± SEM. ***P < 0.001. **E** Total distance traveled by 6-month-old mice in both open and closed arms in the plus-maze test. Data are presented as means ± SEM. *P < 0.05. The control group is Camk2a-tTA mouse. Male (control: n = 5, rTg4510: n = 4) and female (control: n = 5, rTg4510: n = 4–5) mice

rTg4510 mice express a form of human tau containing the P301L mutation, which is associated with AD and frontotemporal dementia. We attempted to identify age-dependent behavior impairment in rTg4510 mice of different ages (Fig. 1A). Because transgene expression is driven by the CaMK2 promoter, the phosphorylated tau is mainly expressed in the cortex and hippocampus [14]. We aimed to confirm that total tau levels and phosphorylated tau levels were increased both before and after our behavioral tests. Therefore, we used western blotting to evaluate the levels of total tau and phosphorylated tau in the cortex and hippocampus of 3- and 6-month-old rTg4510 mice, compared with those in the control group (Camk2a-tTA mouse). The levels of total tau, referred to as Tau5, were increased in the cortex and hippocampus in both age groups of rTg4510 mice (Supplemental Fig. 1). The formation of phosphorylation clusters (such as S214 and S404) induces conformational changes in tau proteins, thereby contributing to the development of pathological features [16, 17]. As we predicted, the phosphorylation of S214 and S404 in tau was also increased in the cortex and hippocampus of 3- and 6-month-old rTg4510 mice (Supplemental Fig. 1). Next, we evaluated behavioral impairment in 4- and 6-month-old rTg4510 mice. Nest building is a spontaneous behavior that has been proposed to represent activities of daily living and well-being in mice and is useful for research on cognitive loss, BPSD, and abolishing daily living activities in AD [18]. In the nest-building test, the nest scores of mice at 4 and 6 months of age were significantly lower than those in the corresponding control groups (Figs. 1B and 2A).

The Y-maze test is used to assess short-term memory in rodents, and spontaneous alternation is used to represent spatial working memory [19]. In the Y-maze test, significantly lower spontaneous alternations of mice that were 6 months old, but not 4 months old, demonstrated impairment of working memory compared with the corresponding control groups (Figs. 1C and 2B). The elevated plus maze can be used to assay anxiety-related behavior in rodents and risk-taking behavior in AD model mice [20, 21]. Both the 4- and 6-month-old rTg4510 mice spent more time in the open arm (Figs. 1D and 2C) than in the closed arm (Figs. 1E and 2D) compared with the corresponding control groups. The total distance in 4-month-olds was the same as that in the corresponding control group, indicating that locomotor activity in the plus-maze test was not changed by the expression of human tau containing the P301L mutation (Fig. 1F). These results suggest that in contrast with control mice, 4-month-old rTg4510 mice exhibited greater levels of risk-taking and exploratory behaviors.

AD patients show body weight loss; thus, we compared the body weights of 4- and 6-month-old rTg4510 mice with those of the corresponding control groups (Supplemental Figs. 2B and 3B) [22]. Consistent with a previous study [23], 6-month-old rTg4510 mice displayed lower body weight than the relevant control group (Supplemental Fig. 3B). Compared with control mice, 6-month-old rTg4510 mice demonstrated normal locomotor activity in the home cage (Supplemental Fig. 3A), and while they showed no change in food intake, they exhibited increased water intake (Supplemental Figs. 3C and D).

**Fig. 3:**
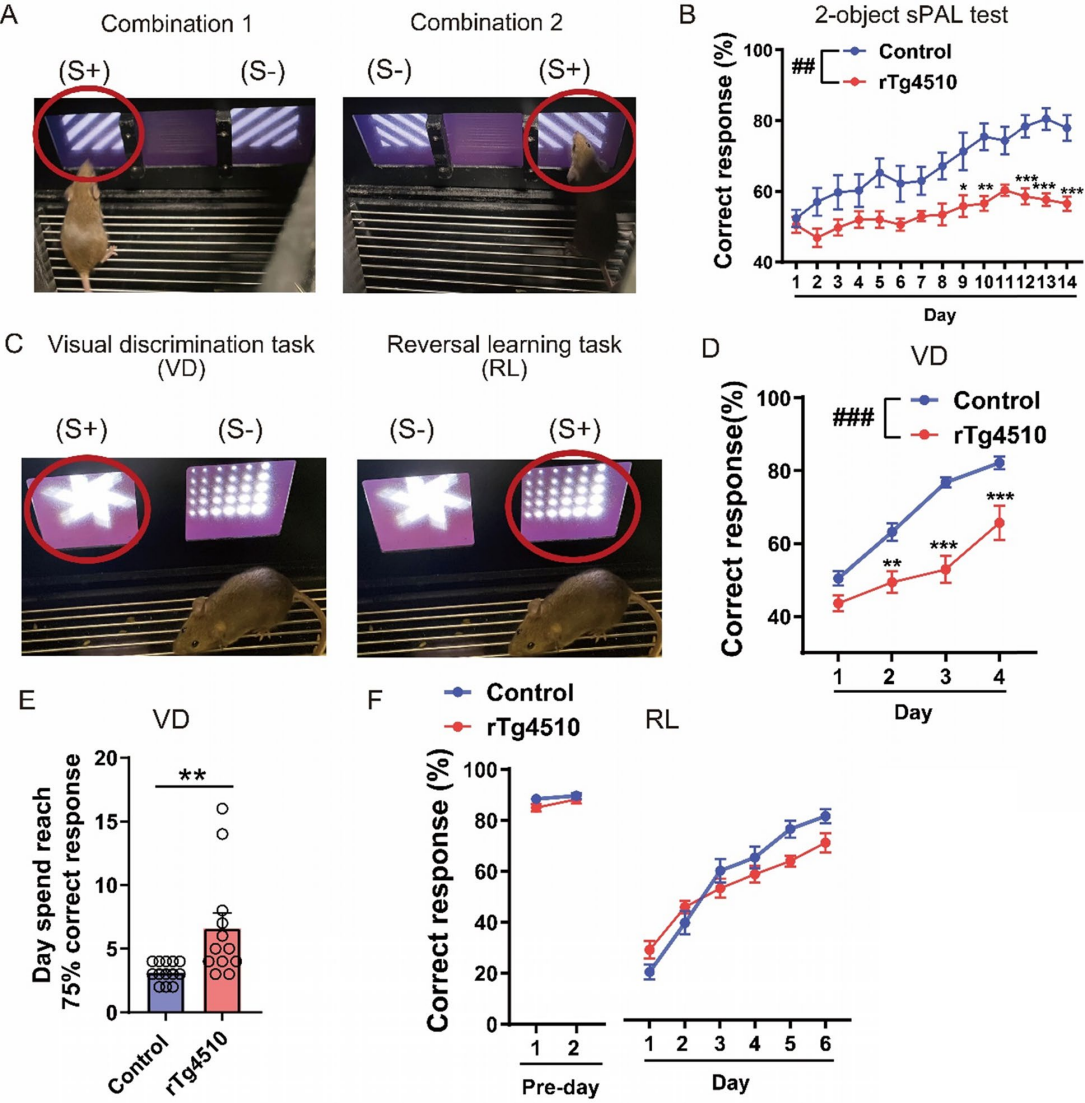
rTg4510 mice displayed impaired associative memory in the two-object sPAL task, whereas in the VD and RL tasks, they exhibited impaired learning but showed no changes in cognitive flexibility. **A** Diagrams representing two combinations of stimuli in the two-object sPAL test. **B** Performance during the 14-day acquisition session of the two-object sPAL test in all mice (control: female n = 3, male n = 6; rTg4510: female n = 4, male n = 4). Data are presented as means ± SEM. *P < 0.05 versus control on day 9, **P < 0.01 versus control on day 10, ***P < 0.001 versus control on day 12 and day 14, ***P < 0.001 versus control on day 13, ^##^*P* < 0.01 versus control. **C** Two types of stimuli were used in the VD and RL tests. **D** Performance during the 4-day acquisition phase of the VD test in all mice (control: female n = 6, male n = 7; rTg4510: female n = 7, male n = 5). Data are presented as means ± SEM. **P < 0.01 versus control on day 2, ***P < 0.001 versus control on day 4, ***P < 0.001 versus control on day 3, ^###^*P* < 0.001 versus control. **E** Days required to reach a 75% correct response rate in the VD test in all mice (control: female n = 6, male n = 7; rTg4510: female n = 7, male n = 5). Data are presented as means ± SEM. **P < 0.01. **F** Performance during the 6-day acquisition phase in the RL test in all mice (control: female n = 5, male n = 7; rTg4510: female n = 3, male n = 5). Data are presented as means ± SEM. The control group is Camk2a-tTA mouse. sPAL, same paired-associate learning; VD, visual discrimination; RL, reversal learning

### rTg4510 mice display impaired associative memory and learning, but show no changes in cognitive flexibility

Paired-associate learning tasks test object-in-place associative memory, and visual discrimination (VD) and reversal learning (RL) tasks measure cognitive flexibility, making them useful for the diagnosis of AD [24]. To study corticohippocampal–dependent object-location memory, we subjected 4- to 5-month-old rTg4510 mice to a two-object same paired-associate learning (sPAL) test [25]. During 14 days of training, control mice learned more quickly than rTg4510 mice, indicating impaired cortico-hippocampal function in the rTg4510 mice (Fig. 3B). The rTg4510 mice showed impairment in object-location memory (Fig. 3B). We next examined deficits in cognitive flexibility in 4–5-month-old rTg4510 mice using VD and RL tasks [26]. The VD task, which was performed to assess discriminative learning and memory [27], depends on the intact function of the corticostriatal circuitry [28, 29]. rTg4510 mice displayed poor discriminative learning and memory performance compared to the control group (Fig. 3D), and needed more days to reach 75% correct response (Fig. 3E). Next, after the threshold (greater than 80% accuracy on two consecutive days) was reached on the Pre-day, RL task evaluated choice shifting and choice learning (Fig. 3F) [29]. There was no difference in the relearning performance between rTg4510 and control mice.

### The cortex of rTg4510 mice shows gene expression changes in glial cells, along with immunostaining evidence of gliosis

**Fig. 4:**
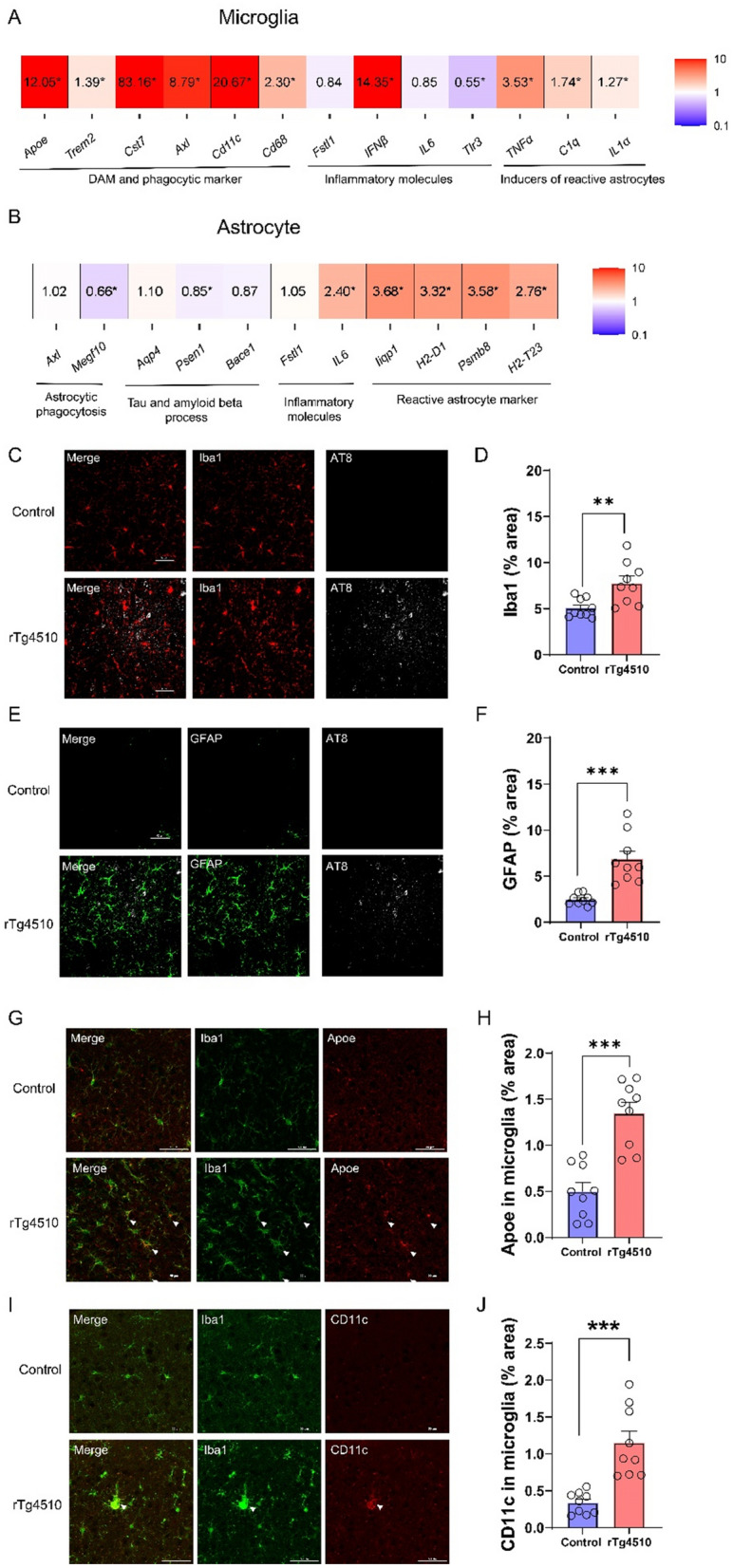
rTg4510 mice exhibited altered gene expressions in both microglia and astrocytes, and immunostaining evidence of gliosis associated with abnormal behavior. **A** Differential gene expression observed in microglia of 6-month-old rTg4510 mice. (control: female n = 1, male n = 4; rTg4510: female n = 2, male n = 3). Data are presented as means ± SEM. *P < 0.05 versus control. **B** Differential gene expression observed in astrocytes of 6-month-old rTg4510 mice (control: female n = 1, male n = 4; rTg4510: female n = 2, male n = 3). Data are presented as means ± SEM. *P < 0.05 versus control. **C** Representative images showing Iba-1 staining (red) in the cortex of rTg4510 and control mice. Scale, 50 µm. **D** Semi-quantification of the Iba1 signal in the cortex of rTg4510 and control mice. Positive signals of cells and processes are presented as % areas (control: female n = 1, male n = 2; rTg4510: female n = 1, male n = 2). ROI in each of three mice per group. Data are presented as means ± SEM. **P < 0.01. **E** Representative images showing GFAP staining (green) in the cortex of rTg4510 and control mice. Scale, 50 µm. **F** Semi-quantification of the GFAP signal in the cortex of rTg4510 and control mice. Positive signals of cells and processes are presented as % areas (control: female n = 1, male n = 2; rTg4510: female n = 1, male n = 2). ROI in each of three mice per group. Data are presented as means ± SEM. ***P < 0.001. **G** Representative images showing Iba1 staining (green) and Apoe staining (red) in the cortex of rTg4510 and control mice. Scale, 50 µm. **H** Semi-quantification of the Apoe signal in the cortex of rTg4510 and control mice. Positive signals of cells and processes are presented as % areas (control: female n = 1, male n = 2; rTg4510: female n = 1, male n = 2). ROI in each of three mice per group. **I** Representative images showing Iba1 staining (green) and CD11c staining (red) in the cortex of rTg4510 and control mice. Scale, 50 µm. ROI in each of three mice per group. Data are presented as means ± SEM. ***P < 0.001. **J** Semi-quantification of the Cd11c signal in the cortex of rTg4510 and control mice. Positive signals of cells and processes are presented as % areas (control: female n = 1, male n = 2; rTg4510: female n = 1, male n = 2). ROI in each of three mice per group. Data are presented as means ± SEM. ***P < 0.001. The control group is Camk2a-tTA mouse. GFAP, glial fibrillary acidic protein; Iba1, microglia/macrophage-specific calcium-binding protein; Apoe, apolipoprotein E protein; CD11c, integrin alpha X protein

Next, to investigate changes in gene expressions of microglia and astrocytes in the cortex of rTg4510 mice, we isolated glial cells using magnetic-activated cell sorting (MACS) and assessed gene expression using qPCR. MACS was performed on cortical tissue from 6-month-old mice to capture glial gene expression changes that coincided with significant behavioral impairment. A previous report suggests that the loss of homeostatic microglial function is associated with the extent of neuronal cell loss and indicates a correlation between glial phenotypes and the severity of neurodegeneration [30]. Therefore, we first aimed to examine glial cell phenotypes and selected candidate genes of DAM and phagocytic markers (*Apoe*, *Trem2*, *Cst7*, *Axl*, *Cd11c,* and *Cd68*) [31, 32], inflammatory molecules (*Fstl1, Tlr3, Il-6,* and *Ifnβ*) [33–36], inducers of reactive astrocytes (*Tnfα, C1q,* and *Il-1α*) [37], reactive astrocyte markers (*Iigq1, H2-d1, Psmb8,* and *H2-t23*) [37], astrocyte-specific phagocytic receptor (*Megf10*) [38, 39], tau clearance (*Aqp4*) [40], and amyloid secretion (*Psen1* and *Bace1*) [41, 42]. In isolated microglia, the mRNA expression levels of most candidate genes were uniformly upregulated in rTg4510 mice (Fig. 4A). Of note, mRNA expression levels of *Trem2, Cd68, TNFα, C1q,* and *IL-1α* in isolated microglia from rTg4510 mice were increased compared with the control group. Moreover, *Apoe*, *Cst7*, *Axl*, *Cd11c,* and *Ifnβ* in isolated microglia from rTg4510 mice were drastically increased compared with the control group. By contrast, *Tlr3* mRNA expression was decreased, while *Fstl1* and *Il-6* mRNA expressions were unchanged in isolated microglia from rTg4510 mice.

In isolated astrocytes, the mRNA expression levels of *Il-6*, *Iigp1*, *H2-d1*, *Psmb8*, and *H2-t23* were significantly increased in rTg4510 mice compared with controls, while those of *Axl*, *Aqp4*, *Bace1*, and *Fstl1* were unchanged (Fig. 4B). Interestingly, the expressions of *Megf10* and *Psen1* were decreased in isolated astrocytes from rTg4510 mice. These results suggest that the environment involved in glial cell maintenance of homeostasis in the brain was significantly different in 6-month-old rTg4510 mice than in controls, leading to abnormal behaviors in the former. In fact, expressions of the microglial marker Iba1 and the astrocytic marker GFAP were also increased in the cortex of rTg4510 mice (Fig. 4C–F), suggesting that both glial cell types were activated. To confirm protein expression levels, we examined the expression of the DAM-associated markers Apoe and Cd11c in the cortex of rTg4510 mice using immunostaining. Both Apoe and Cd11c protein levels were significantly elevated in rTg4510 mice compared to those in the control mice (Fig. 4G–J). These results support the upregulation of DAM-related genes at the protein level and suggest the activation of disease-associated microglia in this tauopathy model.

### Abnormal behavior in rTg4510 mice may be associated with specific gene expression patterns

**Fig. 5:**
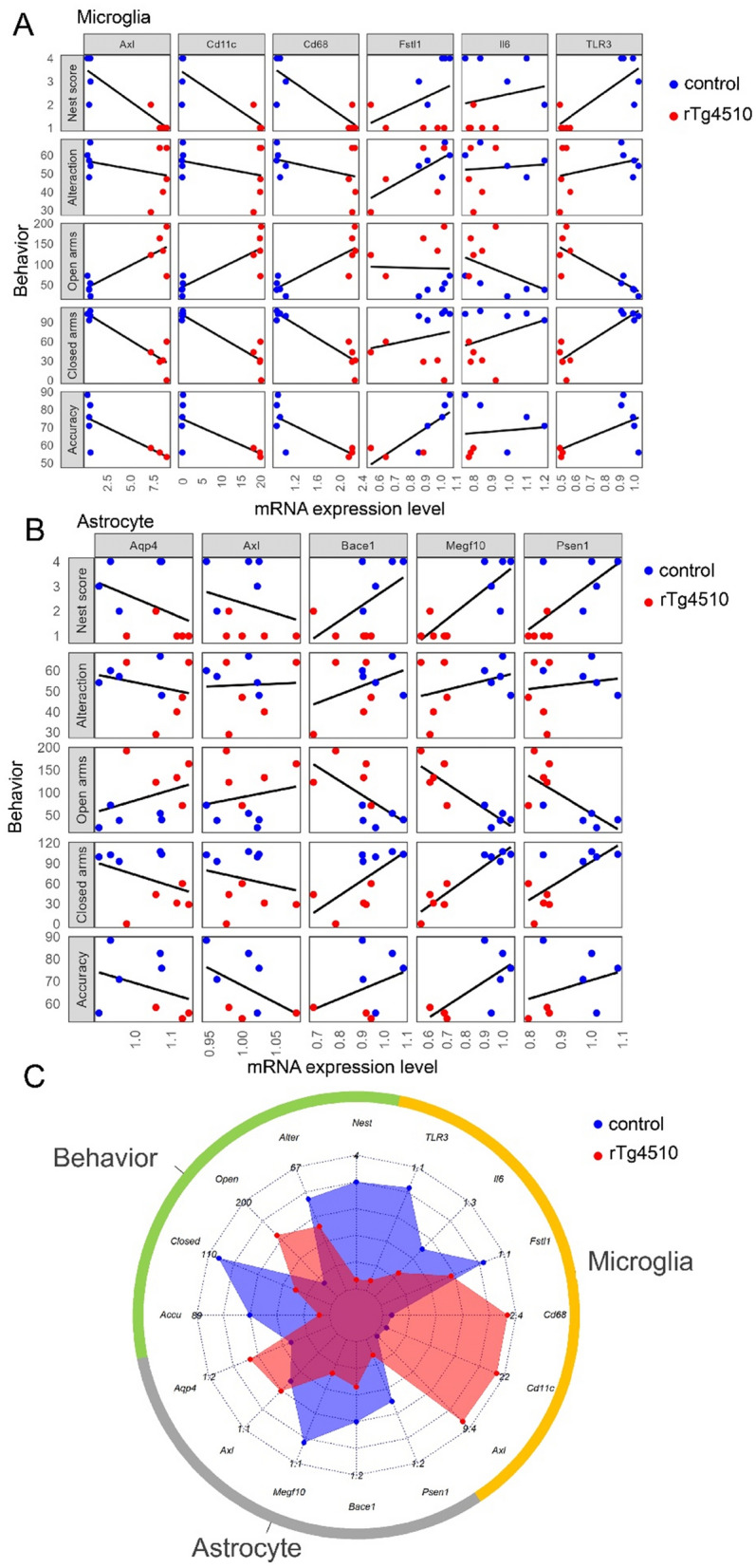
Regression analysis of gene expressions and behavioral changes. **A** Regression analysis was conducted to investigate the relationship between microglial gene expression changes and behavioral alterations in rTg4510 and control mice. **B** Regression analysis was conducted to investigate the relationship between astrocytic gene expression changes and behavioral alterations in rTg4510 and control mice. **C** Radar chart shows changes in gene expressions and behaviors in rTg4510 and control mice. In the radar chart, the numbers represent the values from each behavioral test as well as the gene expression levels. Blue indicates average values for the control group, whereas red indicates average values for the rTg4510 group

We have confirmed that glial phenotypes have changed in our mice model, however, the impact of the expressions of various genes on behavior change is unknown. Therefore, on the basis of the aforementioned findings, we aimed to define a potential ethological index and identify specific gliosis-related factors to track disease progression in rTg4510 mice. We utilized regression analysis, which is used to infer causal relationships between independent and dependent factors [43], to analyze all behavioral and gene data of each subgroup of mice during the observation period. In Fig. 5, we show several candidate genes from Fig. 4A and B that may lead to abnormal behaviors.

Due to limitations associated with the coefficient of determination (R^2^), we used the adjusted coefficient of determination (adjusted R^2^) to assess how gene expression differences between rTg4510 mice and control mice correlated with behavioral changes. Regression analysis of microglial gene expressions revealed high correlations (R^2^ > 0.7) between *Axl, Cd11c*, and *Cd68* gene expression differences and both the nesting score and closed-arm time in the plus-maze test, and between *Tlr3* gene expression differences and closed-arm time (Fig. 5A). Regarding *Axl, Cd11c,* and *Cd68*, there was a positive correlation with open-arm time (adjusted R^2^ = 0.63, *P* = 0.004; adjusted R^2^ = 0.653, *P* = 0.003; adjusted R^2^ = 0.653, *P* = 0.003; respectively), and a negative correlation with nesting score (adjusted R^2^ = 0.758, *P* < 0.001; adjusted R^2^ = 0.736, *P* < 0.001; adjusted R^2^ = 0.72, *P* = 0.001; respectively), closed-arm time (adjusted R^2^ = 0.84, *P* < 0.001; adjusted R^2^ = 0.843, *P* < 0.001; adjusted R^2^ = 0.854, *P* < 0.001; respectively), and sPAL accuracy (adjusted R^2^ = 0.462, *P* = 0.038; adjusted R^2^ = 0.432, *P* = 0.046; adjusted R^2^ = 0.495, *P* = 0.031; respectively). Regarding *Fstl1*, there was a positive correlation with Y-maze test alternation (adjusted R^2^ = 0.381, *P* = 0.031) and sPAL accuracy (adjusted R^2^ = 0.545, *P* = 0.022). Regarding *Tlr3*, there was a positive correlation with nesting score (adjusted R^2^ = 0.607, *P* = 0.005), open-arm time (adjusted R^2^ = 0.641, *P* = 0.003), and closed-arm time (adjusted R^2^ = 0.757, *P* < 0.001). *Il6* expression differences were not correlated with any behavioral changes.

Regression analysis of astroglial gene expressions revealed high correlations between *Megf10* gene expression differences and several behavioral indexes (Fig. 5B). The difference in astrocyte *Megf10* expression between rTg4510 mice and control mice was significantly related to nesting score (adjusted R^2^ = 0.636, *P* = 0.003), open-arm time (adjusted R^2^ = 0.734, *P* < 0.001), and closed-arm time (adjusted R^2^ = 0.877, *P* < 0.001). Interestingly, although *Axl* and *Megf10* are both involved in phagocytosis, the patterns of their correlations with the behavioral indices differed. *Bace1* showed a positive correlation with closed-arm time (adjusted R^2^ = 0.345, *P* = 0.043). *Psen1* showed a positive correlation with nesting score (adjusted R^2^ = 0.407, *P* = 0.028) and closed-arm time (adjusted R^2^ = 0.457, *P* = 0.019), a negative correlation with open-arm time (adjusted R^2^ = 0.434, *P* = 0.023). Statistical data were shown in supplemental Table 3.

Finally, we compared gene expression differences with behavioral data using a radar chart (Fig. 5C). Together our results show that the properties of astrocytes and microglia are different in rTg4510 mice than in control mice.

## Discussion

Our findings demonstrate pronounced behavioral deficits in rTg4510 mice, including impaired executive function (such as that involved in nest building) and increased risk-taking behavior. These phenotypes are the same as those identified in previous reports of cognitive impairment and anxiety-like behaviors in tauopathy models [44, 45], supporting the relevance of rTg4510 mice for studying behavioral outcomes related to tau pathology. A key contribution of this study is the identification of glial activation and its potential role in exacerbating tau-induced neurotoxicity. Upregulated genes linked to phagocytosis and inflammatory pathways were detected in the prefrontal cortex, suggesting that glial cells are not merely bystanders but active participants in disease progression. This aligns with evidence from human tauopathies, where microglial and astrocytic activation correlates with disease severity and cognitive decline [46]. Interestingly, glial activation may have dual roles: promoting the clearance of pathological tau aggregates while simultaneously amplifying neuroinflammation that contributes to neuronal loss. Future studies using pharmacological or genetic modulation of glial activity in rTg4510 mice could help clarify these two functions.

The emergence of hyperphosphorylated human tau species beginning at 2.5 months of age is associated with gliosis, brain atrophy, and synaptic loss in the rTg4510 mouse model [47]. In this study, we conducted behavioral tests in which proper performance required normal functioning of the cortical and hippocampal regions and observed several abnormal behaviors in rTg4510 mice. Nest building is a spontaneous home-cage behavior that has been proposed to represent daily life activities in mice [48] and is widely used as a behavioral measurement in the AD mouse model [18, 49]. Cortico-hippocampal neurons play an important role in nest-building behavior, and this behavior is impaired by hippocampal damage in mice [50–53]. We found impairments of nest-building behavior in rTg4510 mice, which indicates the dysfunction of cortico-hippocampal neurons (Figs. 1 and 2).

The hippocampus is involved in the working memory required to perform the Y-maze test [54–56]. In a previous study, 4-month-old rTg4510 mice exhibited a deficit in the Y-maze test, while 6-month-old rTg4510 mice did not because they developed stereotypic behavior [57]. In this study, however, we found no differences in this test between control and rTg4510 mice at 4 months of age (Fig. 1C). We speculate that the control mice may have had some behavioral deficits, because neuronal loss, neurotoxicity, and behavioral abnormalities have previously been found in mice under single transgenic tetracycline transactivator (tTA) control [58–60]. Additionally, the elevated expression of hTau driven by the tTA system may introduce artificial toxicities that are not reflective of physiological tauopathy. These limitations highlight the need to validate our findings across additional models, such as those with patient-derived tau seeding or physiological levels of tau expression [46]. We found that working memory impairment in 6-month-old rTg4510 mice, indicating more severe behavioral deficits in 6-month-old rTg4510 mice than in 4-month-old rTg4510 mice.

In addition to working memory, we used the elevated plus maze to assess exploratory and risk-taking behaviors. Compared with control mice, both 4- and 6-month-old rTg4510 mice spent significantly less time in the closed arm and significantly more time in the open arm, whereas 4-month-old rTg4510 mice demonstrated no significant difference compared with controls in terms of total distance traveled. These results suggest that 4-month-old rTg4510 mice exhibited not only emotional dysfunction but also impaired exploratory and risk-taking behaviors [20, 21]. As rTg4510 mice age, tau pathology leads to progressive motor deficits, particularly at around 6 months [61]. In this study, 6-month-old rTg4510 mice exhibited an increase in open-arm time alongside a reduction in total distance traveled. This seemingly contradictory pattern highlights a complex interplay between impaired risk-taking behavior and motor impairments in 6-month-old rTg4510 mice.

Next, we assessed the performance of rTg4510 mice in the two-object sPAL test. Object-location memory and proper functioning of the dorsal hippocampal CA1 region are necessary for this test [25]. 4-month-old rTg4510 mice exhibited intact working memory but significantly impaired object-location memory. Taken together with our previous report [8] that conventional behavioral tests may fail to detect cognitive impairments, whereas touchscreen-based tasks are more sensitive. This highlights the utility of touchscreen-based behavioral testing for reliably detecting early cognitive deficits in AD models.

We also assessed the performance of rTg4510 mice in the touchscreen-based VD and RL tests (Fig. 3D, E, and F). In the VD test, the performance of rTg4510 mice was impaired (Fig. 3D and E). VD learning requires perceptual learning and memory processing, both of which involve interactions between multiple cortical areas [62]. In rTg4510 mice, structural abnormalities in the cortex from 4 to 13 months of age result from tau-related cell death in cortical neurons [63]. However, in the RL test, the performance of rTg4510 mice was the same as that of the control group. The RL test assesses behavioral flexibility in learning, which is associated with the prefrontal cortex and striatum [63]. In our previous study, we investigated some performance in App^NL−G−F/NL−G−F^ knock-in mice by introducing three familial AD-associated mutations at the endogenous mouse App locus [8]. These mice constitute a new AD mouse model characterized by overproduction of Aβ42 without overexpression of amyloid precursor protein; they revealed normal cognitive flexibility in the RL test. This finding, together with the demonstration that AD patients show normal cognitive flexibility [64], indicates that neuronal damage induced by tau and amyloid beta may not severely impact the circuits critical for cognitive flexibility.

In gene expression analyses of isolated microglia and astrocytes from rTg4510 mice (Fig. 4), we found abnormalities of mRNA expression levels in both cell types. Apoe is the primary cholesterol carrier in the brain, and Trem2 promotes microglia survival, proliferation, and phagocytosis, making the latter important for cell viability and normal immune functions in the brain [65]. Apoe and Trem2 are associated with the AD pathway, which is upregulated in microglia cells [66]. In our results, phagocytosis-related genes in microglia, such as *Apoe* and *Trem2*, showed significantly increased expression in isolated microglia from rTg4510 mice. Apoe is also involved in the loss of homeostatic microglial function and is responsible for driving neurodegeneration [67]. Our data are consistent with previous reports that markers of DAM, such as *Apoe, Trem2, Cst7*, and *Axl* [31, 32], and those of activated phagocytosis in macrophages and microglia, such as *Cd68* and *Cd11c* [68], are upregulated in rTg4510 mice. The observed upregulation of Apoe and Cd11c protein levels is consistent with previous reports on DAM activation in neurodegenerative models and human AD brain tissues [30]. Abnormal activated microglia and also loss of homeostatic microglia and macrophages may be one of the reasons for progressive neuronal loss in the cortex (Supplemental Figs. 4A and B), which in turn leads to abnormal behavior. In addition, our regression analysis showed that altered gene expression in isolated microglia from rTg4510 mice was correlated with behavioral changes (Fig. 5). In particular, our data indicated that microglial changes in *Axl, Cd11c,* and *Cd68* expression were correlated with the nesting score, closed- and open-arm times, and sPAL accuracy (Fig. 5A). Therefore, these molecules may play a role in abnormal behaviors, such as those related to daily life activities, BPSD, risk-taking, exploratory functioning, and cognitive impairment. The difference in microglial *Tlr3* expression between rTg4510 and control mice was correlated with impairment in nest-building behavior (Fig. 5A); therefore, the expression of this gene may also be involved in daily life activities and BPSD. Thus, our data are consistent with the finding that AD pathologies, such as neuronal loss, may result from dysfunctional microglial phagocytosis [69]. Increased expressions of reactive astrocytic markers such as *H2-d1, Psmb8*, and *H2-T23* in isolated astrocytes from our rTg4510 mice were also associated with behavioral impairments (Fig. 5B). Because the mRNA expression levels of genes that induce reactive astrocytes (*Tnfα, C1q,* and *Il-1α*) were increased in microglia, and those of reactive astrocytic markers (*Iigq1, H2-d1, Psmb8,* and *H2-t23*) were increased in astrocytes, reactive astrocytes may be induced by these DAM-derived inducers (*Tnfα, C1q,* and *Il-1α*). Moreover, we showed that astrocytic *Megf10* expression correlated with dysfunctional daily life activities, BPSD, and cognitive impairment (Fig. 5B), and astrocytes are involved in synapse pruning and clearance of neuronal debris through the MEGF10/MERTK signaling pathway [70]. Consistent with a previous study [71], the current study did not directly find synaptic loss in 6-month-old rTg4510 mice (Supplemental Fig. 4C–E), and the observed changes in astrocytic gene expression may reflect or contribute to synaptic vulnerability. Future studies combining glial activation with synaptic analyses are critical to determine whether astrocyte-mediated mechanisms play a causal role in synaptic degeneration in tauopathy. Since there was also a correlation between tau production and amyloid beta secretion (the latter mediated by *Bace1* and *Psen1*), increased tau phosphorylation may cause abnormalities in the amyloid production pathway [72–74]. These observations suggest that such physiological abnormalities in glia may accelerate AD symptoms, including not only cognitive dysfunction, but also daily life activities and BPSD.

We also examined neurotoxicity in rTg4510 mice. NeuN-positive cells were reduced in the cortex of 6-month-old rTg4510 mice compared to the controls (Supplemental Fig. 4B). Taken together with a previous report, neurodegeneration occurred in the corticohippocampus of 6-month-old rTg4510 mice [75]. In tauopathy models, complement-mediated abnormal microglial phagocytosis has been implicated in the removal of neurons and synapses [69]. Although direct evidence from rTg4510 mice is limited, several studies using other AD models have demonstrated that inhibiting microglial activation reduces abnormal protein accumulation [74, 75], improves behavioral deficits [76, 77], and prevents neuronal loss [76]. The observed abnormalities in glial function and inflammation may lead to neuronal loss, neuronal degeneration, and behavioral abnormalities. However, the detailed mechanisms remain to be investigated in future studies.

The rTg4510 mice in this study mimicked the features of human non-AD tauopathy. The accumulation of intracellular neurofibrillary tangles (NTFs) is induced by P301L hTau in rTg4510 mice. Moreover, suppression of hTau inhibits neuronal loss and partially reverses cognitive dysfunction without preventing NFTs [76, 77]. The impact of different tau species on cognitive dysfunction remains unclear [78]. We suspect that neuroinflammation contributes to cognitive dysfunction. We explored the interplay between tau pathology, glial activation, and behavioral deficits in rTg4510 mice, a widely used model of tauopathy. However, while this animal model exhibits robust tau aggregation and neurodegeneration, several factors beyond hTau overexpression complicate the interpretation of the results. The possibility of confounding effects arising from transgene insertion at the Fgf14 locus must be acknowledged. Disruption of this gene, critical for neuronal excitability, may independently contribute to some behavioral deficits observed, particularly those involving motor coordination and exploratory behavior [15, 60]. To address the limitations of the rTg4510 model, future research could adopt a comparative approach, examining behavioral and glial phenotypes across different tauopathy models. Incorporating patient-derived tau seeds could provide a more accurate representation of human tau pathology. Additionally, integrating molecular analyses (e.g., single-cell transcriptomics) with behavioral assessments could yield deeper insights into cell-type–specific contributions to neurodegeneration and cognitive decline.

This study showed that touchscreen-based behavioral tests, particularly the two-object sPAL test and VD task, were useful to reveal cognitive function impairments in rTg4510 mice. Few studies have focused on such tests in rTg4510 mice. Moreover, the dysregulation of glial homeostasis may affect neuronal dysfunction, thereby leading to emotional and cognitive deficits in rTg4510 mice. The results of this study indicate a correlation between glial phenotypes and behavioral dysfunction, and provide important evidence to better understand the role of glial dysfunction in the progression of tau-induced neurodegeneration. However, we did not directly assess the correlation between changes in glial gene expression and classical AD pathological markers. Therefore, further studies are warranted.

## Materials and methods

### Mice

We used rTg4510 (TauP301L) mice that express human tau containing the P301L mutation. To generate rTg4510 mice, FVB-Fgf14^Tg(tetO−MAPT*P301L)4510Kha^/JlwsJ mice and Cg-Tg (Camk2a-tTA) 1Mmay/DboJ mice were obtained from the Jackson Laboratory, and crossbred according to a previous report [79]. We used littermate CaMk2a-tTA mice as a control group [14]. All mice were genotyped by PCR amplification of DNA extracted from the tail. Mice were 4 months old at the start of the behavioral assessment. All mice were maintained under a regulated environment with a 12-h light/dark cycle (light from 8:00 AM to 8:00 PM). Food (CE-2, CLEA Japan) and tap water were available ad libitum. All experiments were performed following the Guidelines for Animal Experiments of Nagoya University, the Guiding Principles for the Care and Use of Laboratory Animals (approved by the Japanese Pharmacological Society), and the United States National Institutes of Health Guide for the Care and Use of Laboratory Animals. All experimental procedures were approved by the Institutional Animal Care and Use Committee of Nagoya University (Permit Number: M250107-001, M250108-001). The number of male and female mice used in each experiment was shown in Supplemental Tables 4 and 5.

### Behavioral assessment

#### Nest-building test

According to a previous report [48], we assessed nest-building performance to evaluate daily life activity. We prepared separate test cages in the test room, and each mouse was individually housed with food and water. The environmental condition was the same as that in the housing room. The 4- and 6-month-old rTg4510 and control mice were each given two pieces of 3 cm × 3 cm pressed cotton squares (which mice use to make nests). At 9:00 AM, one mouse was put in the test cage, which contained bedding material (Japan SLC, Inc., Japan) and pressed cotton (Ancare, USA). The cotton was placed in the same position in all cages. The test cage was not moved for the 24-h test period. At 24 h, the nest was scored according to the following scale: Score 1, shredded paper or small squares remained scattered throughout the cage, or the cotton square or twist remained untouched; Score 2, some of the material was constructed into a nest, but over 50% of the material was not used for nest construction, remaining scattered or untouched; Score 3, a noticeable nest was constructed, but several pieces were still scattered; Score 4, almost all the material was used for the nest, but a few pieces remained scattered or were near the nest; Score 5, all material was used to make an identifiable nest [80]. Nesting scores were assessed in 4- and 6-month-old mice: control (4 months: females n = 5, males n = 4; 6 months: females n = 5, males n = 5) and rTg4510 mice (4 months: females n = 5, males n = 4; 6 months: females n = 5, males n = 4).

#### Y-maze test

This test involved a Y-shaped maze with three white, opaque plastic arms, each oriented at a 120° angle from the adjoining arms. A mouse was introduced to the center of the maze, and was allowed to freely explore the three arms for 8 min, after the course of multiple arm entries. For the assessment of spontaneous alternations, alternations were scored manually, with a sequence of arm visits (e.g., A to B to C) without repetition counted as one complete alternation. An incorrect entry was recorded when the animal revisited the first arm after entering a second arm (e.g., A to B to A), Alternation was defined as successive entry into the three arms on overlapping triplet sets. Alternation behavior (%) was calculated as the ratio of actual alternations to possible alternations (defined as the number of arm entries minus two) multiplied by 100 [81]. Y-maze test was conducted on 4- and 6-month-old mice: control (4 months, females n = 5, males n = 4; 6 months, females n = 5, males n = 5) and rTg4510 mice (4 months, females n = 5, males n = 4; 6 months, females n = 5, males n = 4).

#### Plus-maze test

An elevated plus-maze apparatus was placed in a test room. The apparatus consisted of two open arms (25 cm × 5 cm) and two closed arms of the same size with 15-cm-high walls and a central square (5 cm × 5 cm) connecting the arms. The floor of the arms and the central square were elevated to a height of 55 cm above the floor. A mouse was introduced to the center of the maze, and was allowed to freely explore the open and closed arms for 5 min [20]. The times that the animal spent in the open and closed arms were recorded using a camera that was part of the Ethovison system (Noldus, Netherlands). Plus-maze test was conducted on 4- and 6-month-old mice: control (4 months, females n = 5, males n = 4; 6 months, females n = 5, males n = 5) and rTg4510 mice (4 months, females n = 5, males n = 4; 6 months, females n = 4, males n = 4).

#### Two-object same paired-associate learning (sPAL) test

##### Touchscreen apparatus

Touchscreen assessments were conducted within a specialized operant system designed for mice, situated in a sound- and light-controlled enclosure measuring 87 cm × 50 cm × 79 cm (TOP-M1; O’Hara & Co., Ltd., Tokyo, Japan). The touchscreen apparatus was the same as that used in our previous experiment [8]. Within this enclosure, sound disturbances were minimized by a housing containing a light, a ventilation fan producing white noise, and a pair of tone generators. The operant system itself featured a 15-inch touchscreen unit and a dispenser for 10-mg food pellets on the opposite side. A photocell head entry detector and a camera were positioned directly above the chamber. To mitigate unintended responses triggered by tail or body contact with the touchscreen, a customized black plastic barrier with task-specific response windows was installed in front of the screen. In this study, three windows were allocated for the two-object sPAL test, while two windows were designated for visual discrimination (VD) and reversal learning (RL) tasks [25, 26].

##### Pretraining

The pretraining session was also the same as that in our previous experiment [8]. Before the touchscreen tests, mice were food restricted until their body weight reached 85–95% of the body weight they achieved under ad libitum feeding. The mice then underwent the following pretraining exercises according to the protocol in previous studies. (1) Magazine training (1 day, 30 min): mice received a 10-mg food pellet when their head entered the food receptacle. (2) Autoshaping training (1 day, 30 min): a 10-mg food pellet was delivered after a white stimulus contingent disappearance of presentation from all windows. (3) Must touch (2 days, 60 min/120 trials): mice had to touch the touchscreen to receive a food pellet; white stimuli were presented in all windows. (4) Correct touch (2 days, 60 min/120 trials): to receive a food pellet, mice had to touch a stimulus that was randomly presented in only one window; an incorrect response resulted in no food reward. (5) Correct touch error (2 days, 60 min/120 trials): mice had to correctly touch a randomly presented white stimulus to receive a food pellet, in this test, mice were required to reach a correct response rate of greater than 80% for two consecutive days to qualify for the next test. (6) No error must touch (2 days, 60 min/120 trials): mice had to touch the touchscreen displaying place-associated stimuli, but an incorrect response had no effect.

##### Test

In the test session, we modified the previous method [25] and simplified the visual stimuli. We used mask and stimulus dimensions as follows: number of windows, 3; window size, 57 mm × 57 mm; window gaps, 10 mm; floor gap, 25 mm; stimulus size, 53 mm × 53 mm. After pretraining sessions, the two-object sPAL test was performed as previously described [25]. There were two distant windows, and two images (two right oblique lines and two left oblique lines) were used as visual stimuli in this task. Because mice might make more mistakes due to the proximity of the two windows, an empty space was used to separate the two windows. There were two possible combinations (Fig. 3A), each comprising two sets of lines that slanted either upward or downward from left to right. The upward slanting lines on the left in Combination 1 (S +) and the downward slanting lines on the right in Combination 2 (S +) were considered to be the correct responses. Sixty trials were performed using each combination. The task was complete when the mouse finished 120 trials, or when 60 min had elapsed, whichever came first. Four-to-five-month-old mice were used for the sPAL test (control: female n = 3, male n = 6; rTg4510, female n = 4, male n = 4).

#### VD and RL tasks

VD and RL tasks were performed as previously described [8, 26]. In the pretraining sessions, mice had to correctly touch the randomly presented white stimulus to receive a food pellet; for both tasks, these sessions involved magazine training, autoshaping training, must touch (2 days, 60 min/100 trials), correct touch (2 days, 60 min/100 trials), and correct touch error (2 days, 60 min/100 trials, at least 80% correct response). The pretraining setup was the same as that of the two-object sPAL test, but two windows were used instead of three. Mice had previously been trained to select the touchscreen image showing a pair of black-and-white, brightness-matched stimuli, one of which was correct (S +) and the other incorrect (S −) (Fig. 3C). A response to the correct image (S +) resulted in a tone, magazine illumination, and delivery of a single reward pellet. A response to the incorrect image (S −) caused the house light to be turned off. Responding to the incorrect image was recorded as a mistake, which initiated a correction trial. During the correction trial, the incorrect image (S −) was displayed again after a 5-s timeout and a 3-s inter-trial interval (ITI). After the mice reached the VD task criterion (> 80% accuracy on 2 consecutive days), they proceeded to the reversal phase. Mice that failed to reach the VD task criterion after 1 week of training were excluded. During the reversal phase, the two visual stimuli were reversed. In the reversal phase, reward contingencies of (S +) and (S −) (Fig. 3C) were reversed [8, 26]. Four-to five-month-old mice were used for the VD test (control: female, n = 6; male, n = 7; rTg4510, female n = 7, male n = 5). Four-to five-month-old mice were used for the RL test (control: female, n = 5; male, n = 7; rTg4510, female n = 3, male n = 5). In the VD task, nearly all control mice met the VD task criterion. In contrast, only 8 out of 12 rTg4510 mice met the VD task criterion.

#### Locomotor activity and food/water intaken.

Locomotor activity was analyzed continuously for 24 h from 9:00 a.m. to 9:00 a.m. on the following day. In the measurement of locomotor activity, mice were placed individually in a home cage, and locomotor activity was measured every 5 min for 60 min using digital counters with an infrared sensor (NS-DAS-8; Neuroscience, Tokyo, Japan). Locomotor activity was conducted on 4- and 6-month-old mice: control (4 months, females n = 5, males n = 4; 6 months, females n = 3, males n = 5) and rTg4510 mice (4 months, females n = 5, males n = 4; 6 months, females n = 5, males n = 3).

Food and water intaken were measured over a period of three days. The weight of consumed food and water was recorded daily for each mouse. Food and water intaken were measured in 6-month-old mice (control: female n = 5, male n = 4; rTg4510: female n = 5, male n = 4).

### Magnetic-activated cell sorting (MACS)

Microglia and astrocytes were isolated from the cerebral cortices of mice using magnetic-activated cell sorting (MACS), as previously described [30, 82]. In brief, after mice were transcardially perfused with phosphate-buffered saline (PBS) under deep anesthesia, the cerebral cortex was dissociated at 37 °C for 15 min using the Neural Tissue Dissociation Kit-Postnatal Neurons (Miltenyi Biotec, Bergisch-Gladbach, Germany) with a MACS Dissociator (Miltenyi Biotec). To isolate microglia, myelin debris was removed using Myelin Removal Beads II (Miltenyi Biotec), and purified cells were incubated with anti-CD16/CD32 antibodies (Thermo Fisher Scientific, Waltham, MA, USA) for blocking Fc receptors, followed by incubation with anti-CD11b MicroBeads (Miltenyi Biotec). Using MACS, CD11b-positive microglia were isolated with an LS column (Miltenyi Biotec). Astrocyte isolation was performed by incubating astrocyte-containing, CD11b-negative flow-through cells with anti-ACSA2 MicroBeads (Miltenyi Biotec), and then subjected to MACS through the LS column. Six-month-old mice were used (control: female n = 1, male n = 4; rTg4510: female n = 2, male n = 3).

### Quantification of mRNA levels using real-time PCR

The RNeasy Micro Kit (Qiagen) was used to extract total RNA from MACS-isolated microglia and astrocytes. Complementary DNA (cDNA) from MACS-isolated cells was generated by reverse transcription of total RNA (2.5 or 5 ng) using the PrimeScript™ RT reagent Kit (Perfect Real Time) (Takara Bio, Kusatsu, Japan), and 1/50 of the yield was amplified using the SYBR Premix Ex Taq II (Tli RNaseH Plus) (Takara Bio) and the Thermal Cycler Dice Real Time System II or III (Takara Bio). The PCR protocol was as follows: one cycle at 95 °C for 30 s; 40 cycles at 95 °C for 5 s and 60 °C for 30 s; and dissociation stages at 95 °C for 15 s, 60 °C for 30 s, and 95 °C for 15 s. Actb was used for normalization. Primer information was shown in Supplemental Table 1.

### Immunohistochemistry

Mice were deeply anesthetized with isoflurane (MSD Animal Health, USA), and were transcranially perfused with saline followed by 4% paraformaldehyde (PFA) solution. Animals did not respond to noxious stimuli before the perfusion started. For immunohistochemistry, the brains were removed and fixed overnight in 4% PFA and stored in 20% sucrose, followed by 30% sucrose, then embedded in Tissue-Tek O.C.T. compound (Sakura Finetech, Japan) and stored at − 80 °C. Brain slices (20-µm thickness) were washed with PBS containing 0.3% Triton X-100 and blocked at room temperature for 1 h in the presence of 5% normal donkey serum and 5% normal goat serum (Vector Laboratories, Burlingame, CA, USA). The samples were incubated with mouse anti–Phospho-Tau (Ser202, Thr205) monoclonal (AT8) (MN1020, diluted 1:100; Invitrogen, Carlsbad, CA, USA), goat anti–AIF-1/Iba1 (NB100-1028, diluted 1:250; Novus Biologicals, USA), rabbit anti-Iba1 (019–19741, diluted 1:500; Wako), chicken anti–GFAP (ab4674, diluted 1:1000; Abcam, UK), mouse anti–Apoe (ab1906, diluted 1:200; Abcam, UK), hamster anti–mouse Cd11c (550,286, diluted 1:10; BD Biosciences), and mouse anti–NeuN (3,832,727, diluted 1:100; EMD, USA) antibodies at 4 °C overnight. After washing with PBS, the sections were incubated with Alexa Fluor™ 488 goat anti-rabbit (A11008, 1:1000; Invitrogen); goat anti-hamster DyLight594 (405,504,1:500; BioLegend); Alexa Fluor™ 405 donkey anti–mouse (A48257, diluted 1:1000; Invitrogen), Alexa Fluor™ 546 donkey anti–goat (A11056, diluted 1:1000; Invitrogen), Alexa Fluor™ 488 donkey anti–goat (A11055, diluted 1:1000; Invitrogen), Alexa Fluor™ 594 donkey anti–mouse (A21203, diluted 1:1000; Invitrogen), Alexa Fluor™ 488 donkey anti–mouse (A21202, diluted 1:1000; Invitrogen), and Alexa Fluor™ 488 donkey anti–chicken IgG (703–545-155, diluted 1:1000; Jackson ImmunoResearch, USA) secondary antibodies at room temperature for 1 h. For confocal microscopy, images were obtained using TiE-A1R (Nikon, Japan) and analyzed at 20 × magnification. Because MACS involved the cerebral cortex, images of the cerebral cortex of 6-month-old mice were obtained. For quantitative analysis of immunohistochemistry, three regions of interest (ROIs) per slice × three slices in the brain region of the cortex were used in each mouse. Positive Iba1 and GFAP signals are presented as % areas. Immunohistochemical analysis was performed using the open-source software platform ImageJ [83]. Positive signals were defined as those with a signal intensity above the background threshold. Six-month-old mice were used (control: female n = 1, male n = 2; rTg4510, female n = 1, male n = 2). Antibody information was shown in Supplemental Table 2.

### Western blotting

For western blotting of tau, brain samples were homogenized at 4 °C in RIPA buffer (LOT XI1352172; Thermo Fisher Scientific). The homogenate was centrifuged at 13,000 × *g* for 20 min and the resultant supernatant was retained for western blotting. The protein concentration was determined using a DC Protein Assay Kit (Bio‐Rad Laboratories, Hercules, CA, USA), and protein was boiled in sample buffer (2 × Laemmli Sample Buffer; Bio‐Rad), applied to an SDS‐polyacrylamide gel (Any kD Mini-Protean® TGX Precast Gels, Cat. #4,569,036; Bio-Rad), and subsequently transferred to a polyvinylidene difluoride membrane (Trans-Blot® Turbo, Cat. #1,704,156; Bio-Rad). The membrane was incubated with blocking buffer (EveryBlot Blocking Buffer, Cat. #12,010,020; Bio-Rad) for 30 min and then incubated with mouse anti‐Tau monoclonal Antibody (Tau5) (#AHB, diluted 1:500; Invitrogen), rabbit anti–phospho-Tau (Ser404) (#20,194, diluted 1:1000; Cell Signaling Technology, Danvers, MA, USA), rabbit anti–phospho-Tau (Ser214) (#77,348, diluted 1:1000; Cell Signaling), rabbit anti–PSD95(2507S, diluted 1:1000; Cell Signaling), and rabbit anti–synapsin (AB1543P, diluted 1:1000; Millipore) at 4 °C overnight. After incubation with a horseradish peroxidase–conjugated anti‐rabbit antibody and a horseradish peroxidase–conjugated anti‐mouse antibody for 2 h, the immune complex was detected using ECL Plus western blotting detection reagents (GE Healthcare, Chalfont St. Giles, Buckinghamshire, UK). The intensities of the bands on the membranes were analyzed using LuminoGraph I (Atto Instruments, Tokyo, Japan). Three- and six-month-old mice were used: control (3 months, females n = 3, males n = 1; 6 months, females n = 4, males n = 0) and rTg4510 (3 months, females n = 1, males n = 3; 6 months, females n = 4, males n = 0). Information on antibodies was shown in Supplemental Table 2.

### Regression analysis

Regression analysis is a statistical measure that defines the connection between two variables, indicating how they are intertwined. It delineates the impact of changes in one variable on another [43]. Hence, we employed regression analysis using R-Studio [84] to discern the relationship between gene expression levels and behavioral values. The adjusted R^2^ and P values were shown in Supplemental Table 3. As the adjusted R^2^ accounts for model complexity, unlike the standard R^2^, it penalizes the inclusion of non-informative predictors and provides a more accurate measure of model fit, particularly in studies with small sample sizes. Therefore, the adjusted R^2^ was used in the regression analysis.

### Statistical analysis

All data are expressed as means ± SEM. Statistical analyses were performed with GraphPad Prism 7 (RRID: SCR_002798; GraphPad Software, San Diego, CA, USA). Statistical significance (*P* < 0.05) was determined using Student’s t-test for comparisons between two groups, two-way analysis of variance (ANOVA) for multigroup comparisons, or repeated-measures ANOVA. Sidak’s multiple comparisons test and Tukey’s test were used for post hoc comparison when the F value was significant. All statistical data were shown in Supplemental Tables 4 and 5.

## Supplementary Information

Supplementary file 1

Supplementary file 2

## Data Availability

No datasets were generated or analysed during the current study.

## Abbreviations

*Apoe*: Apolipoprotein E
*Aqp4*: Aquaporin-4
*Axl*: AXL receptor tyrosine kinase
*Bace1*: Beta-secretase
*Cst7*: Cystatin F
*Cd11c*: Integrin alpha X
*Cd68*: Macrosialin
*C1q*: Complement component C1q
DAM: Disease-associated microglia
*Fstl1*: Follistatin-like protein 1
*H2-d1*: Histocompatibility 2, D region locus 1
*H2-t23*: Histocompatibility 2 T region locus 23
*Ifnβ*: Interferon-β
*Il-6*: Interleukin-6
*Il-1α*: Interleukin-1 alpha
*Ligp 1*: Interferon-inducible GTPase 5
*Megf10*: Multiple EGF-like domains 10
*Psen*: Presenilin 1
*Psmb8*: Proteasome subunit beta type 8
*Trem2*: Triggering receptor expressed on myeloid cells 2
*Tlr3*: Toll-like receptor 3
*Tnf*α: Tumor necrosis factor alpha
