# Supplementary material for: Abnormal behaviors and glial responses in an animal model of tau pathology
Source: Mol Brain. 2025 Nov 6;18:83. doi: 10.1186/s13041-025-01252-4 (PMC12590737; doi:10.1186/s13041-025-01252-4)
Supplement: Supplementary file 1 [file 13041_2025_1252_MOESM1_ESM.docx]

**Supplemental Fig.1**

**
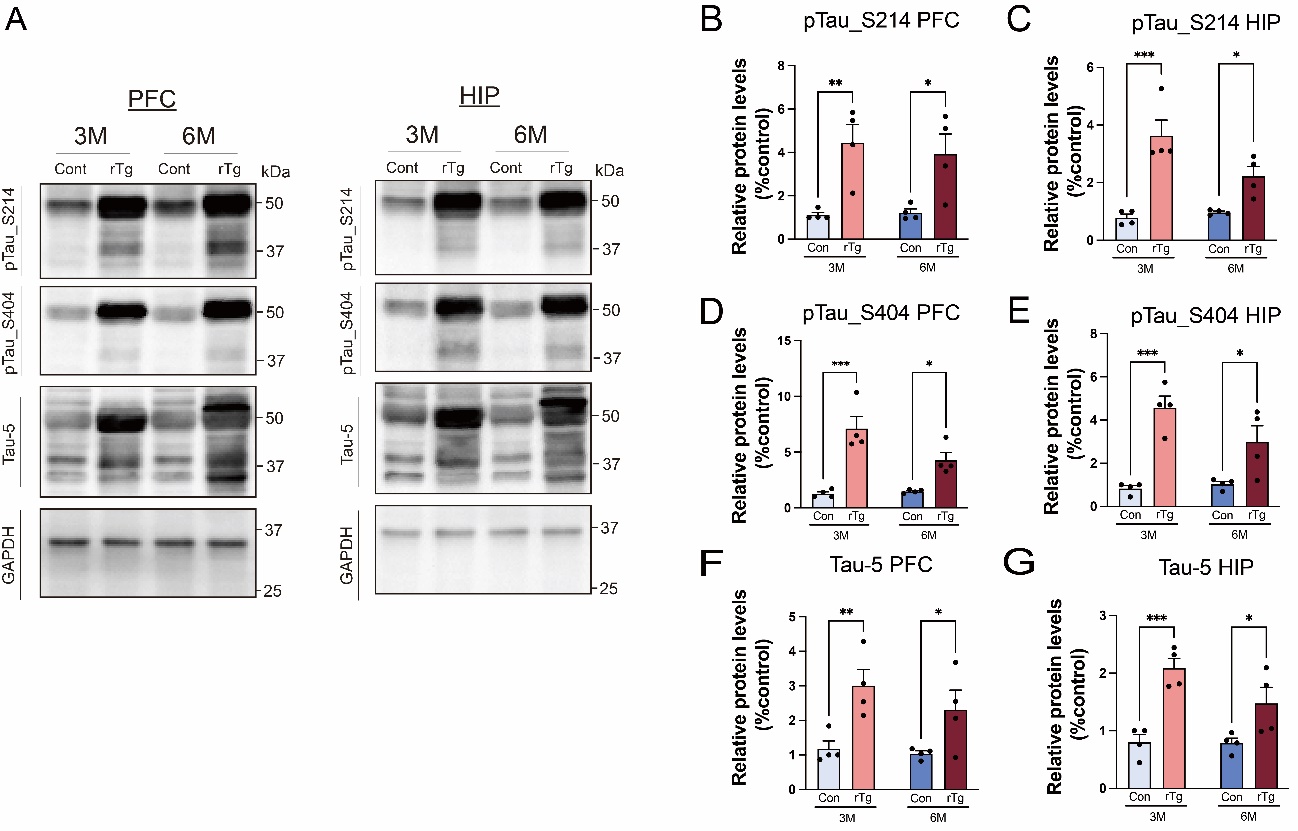
**

**Supplemental Fig 1.** The levels of total tau, phosphorylation of S214 and S404 in tau were increased in the cortex and hippocampus of 3- and 6-month-old rTg451**0** mice. **A,** Elevated levels of phosphorylated tau compared to control mice. **B,** The phosphorylation tau of S214 level from the prefrontal cortex in 3-month-old and 6-month-old control mice and rTg4510 mice (n = 4). Data are presented as means ± SEM. **P < 0.01, *P < 0.05. **C,** The phosphorylation tau of S214 level from the hippocampus in 3-month-old and 6-month-old control mice and rTg4510 mice (n = 4). Data are presented as means ± SEM. ***P < 0.001, *P < 0.05. **D,** The phosphorylation tau of S404 level from the prefrontal cortex in 3-month-old and 6-month-old control mice and rTg4510 mice (n = 4). Data are presented as means ± SEM. ***P < 0.001, *P < 0.05. **E,** The phosphorylation tau of S404 level from the hippocampus in 3-month-old and 6-month-old control mice and rTg4510 mice (n = 4). Data are presented as means ± SEM. ***P < 0.001, *P < 0.05. **F,** The total tau level from the prefrontal cortex in 3-month-old and 6-month-old control mice and rTg4510 mice (n = 4). Data are presented as means ± SEM. **P < 0.01, *P < 0.05. **G,** The total tau level from the hippocampus in 3-month-old and 6-month-old control mice and rTg4510 mice (n = 4). Data are presented as means ± SEM.***P < 0.001, *P < 0.05. PFC, prefrontal cortex; HIP, hippocampus.

**Supplemental Fig.2**

**
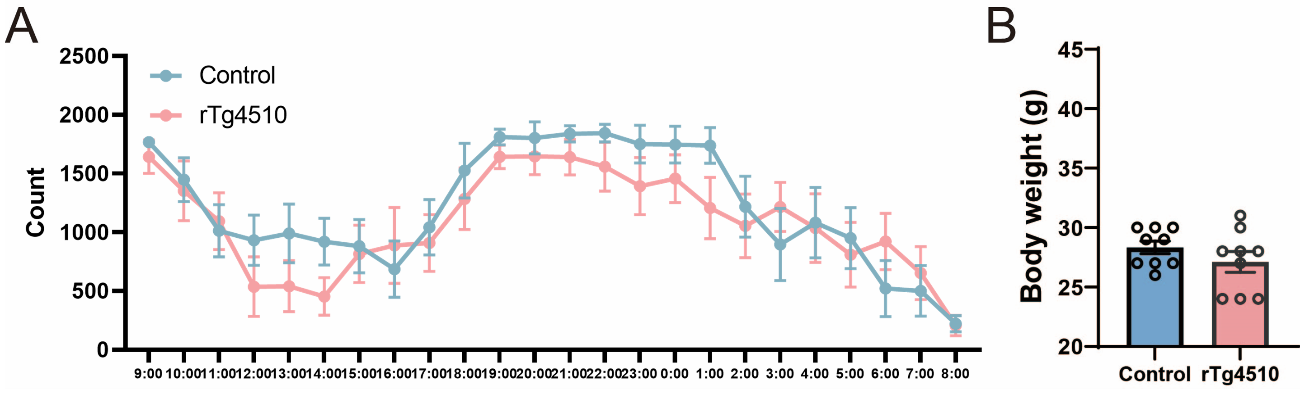
**

**Supplemental Fig 2.** Assessment of locomotor activity in 4-month-old rTg4510 mice was conducted over 24 h to investigate patterns of motor behavior and potential abnormalities. **A,** No changes in motor behavior were observed in rTg4510 mice over 24 h (n = 9). Data are presented as means ± SEM. **B,** rTg4510 mice showed no significant differences in body weight compared with control mice (n = 9). Data are presented as means ± SEM.

**Supplemental Fig.3**

**
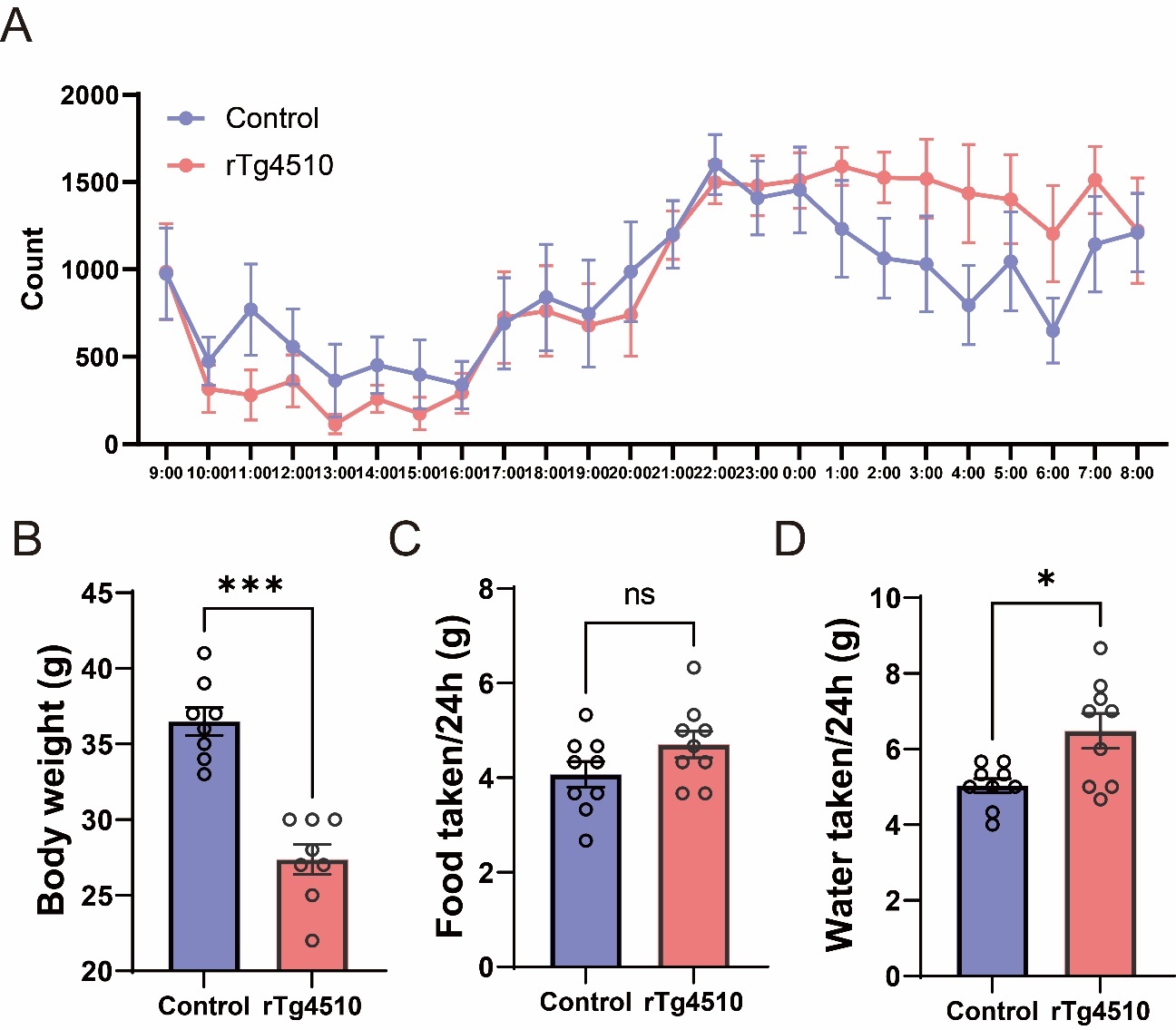
**

**Supplemental Fig 3.** Assessment of locomotor activity in 6-month-old rTg4510 mice was conducted over 24 h to investigate patterns of motor behavior and potential abnormalities. Body weight and 24-h consumption of food and water were also examined. **A,** No changes in motor behavior over 24 h were observed in 6-month-old rTg4510 mice (n = 8). Data are presented as means ± SEM. **B,** Body weight in rTg4510 mice was lower than that in controls (n = 8). Data are presented as means ± SEM. ***P < 0.001. **C,** No differences in 24-h food consumption were observed in rTg4510 mice compared with controls (n = 9). Data are presented as means ± SEM. **D**, rTg4510 mice showed increased 24-h water consumption compared with controls (n = 9). Data are presented as means ± SEM. *P < 0.05.

**Supplemental Fig.4**

**
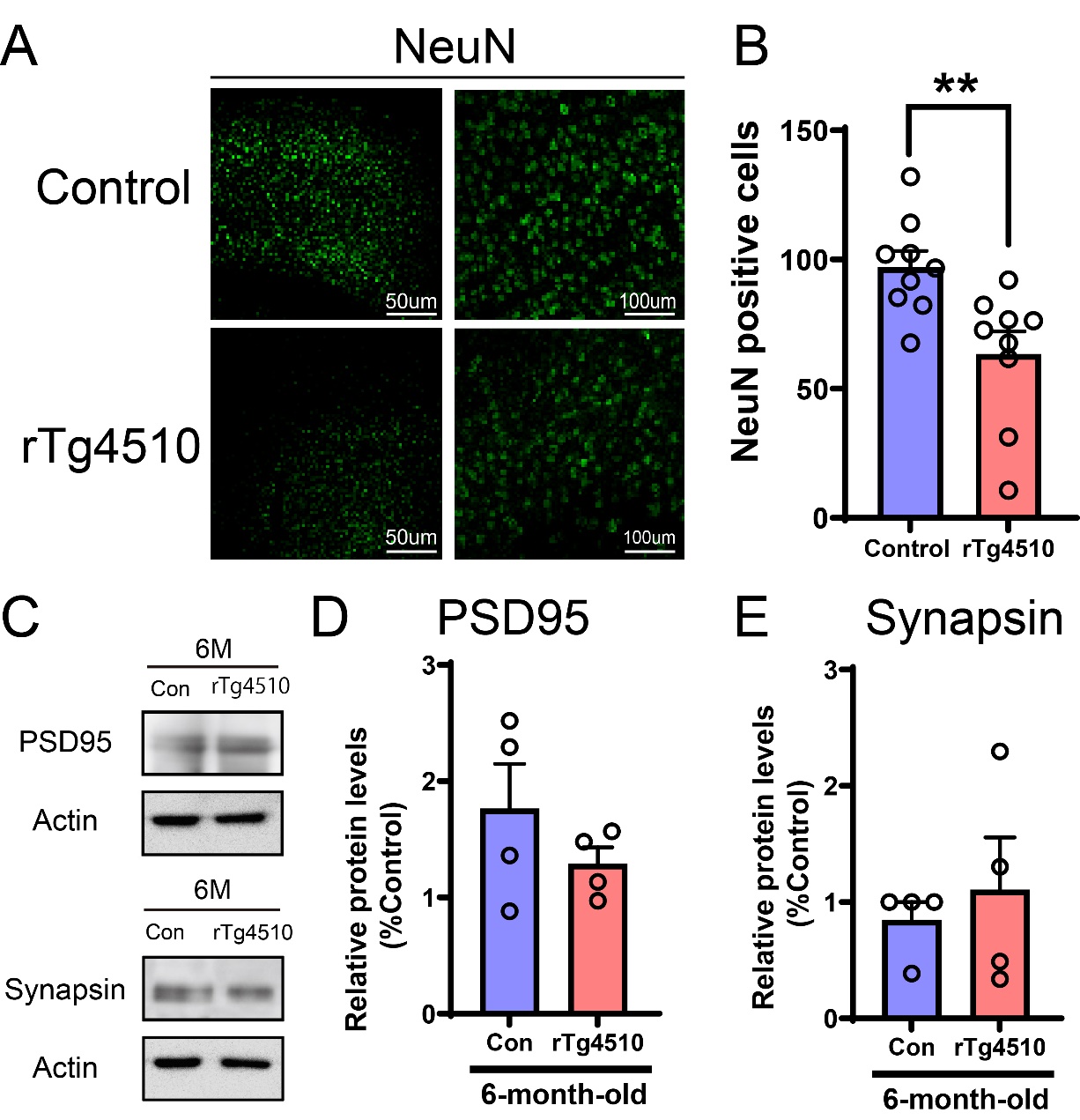
**

**Supplemental Fig 4. A,** Representative images showing neuronal markers (green) in the cortex of rTg4510 and control mice. Scale, 50-100µm. **B,** Number of NeuN positive cells in the cortex of rTg4510 and control mice (control: n = 3; rTg4510: n = 3). ROI in each of three mice per group. **C,** Representative images of PSD95 and synapsin were shown. Levels of PSD95 (D) and synapsin (E) were measured in the prefrontal cortex of 6-month-old control mice and rTg4510 mice (n = 4). Data are presented as mean ± SEM.
