# Supplementary material for: Abnormal behaviors and glial responses in an animal model of tau pathology
Source: Mol Brain. 2025 Nov 6;18:83. doi: 10.1186/s13041-025-01252-4 (PMC12590737; doi:10.1186/s13041-025-01252-4)
Supplement: Supplementary file 2 [file 13041_2025_1252_MOESM2_ESM.docx]

| **Supplemental table 1. Primer information** | | | |
| --- | --- | --- | --- |
| **Target gene** | **Forward** | **Reverse** | **Reference** |
| *Apoe* | GAACCGCTTCTGGGATTACCTG | GCCTTTACTTCCGTCATAGTGTC | [doi: 10.1186/s40478-020-01099-x](https://doi.org/10.1186%2Fs40478-020-01099-x) |
| *Axl* | 5-ATGGCCGACATTGCCAGTG-3 | 5-CGGTAGTAATCCCCGTTGTAGA-3 | doi: 10.1002/glia.23214 |
| *Aqp4* | 5’-AACCTCACCGCTGGCCATGGGCTCCTG-3 | 5’-TACGGAAGACAATACCTCTCCCGAAGAGTC-3 | doi: 10.1186/s40478-020-00936-3. |
| *Bace* | 5’-AGAGGCAGCTTTGTGGAGATGGTGGACAACCTGAG-3’ | 5’-ATGTTGGGAATGTGGGTCTGCTTCACCAGGGAGTC-3’ | doi: 10.1186/s40478-020-00936-3. |
| *C1q* | CTCAGGGATGGCTGGTGGCC | CCTTTGAGACCCGGCCTCCCC | doi: 10.1093/cvr/cvab289 |
| *Cd11c* | CTGGATAGCCTTTCTTCTGCTG | GCACACTGTGTCCGAACTCA | [doi: 10.1186/s40478-020-01099-x](https://doi.org/10.1186%2Fs40478-020-01099-x) |
| *Cd68* | TGACAAGGGACACTTCGGG | GGAGGACCAGGCCAATGAT | doi: 10.1016/j.bbadis.2016.07.007. |
| *Cst7* | ACCAATAACCCAGGAGTGCTTA | TGACCCAGACTTCAGAGTAGCA | [doi: 10.7554/eLife.85279](https://doi.org/10.7554%2FeLife.85279) |
| *Fstl1* | CACCAGGGCACAGCAGAAA | GTGCTCTGTGCCTCTTCTTAGATCT | doi: 10.1186/s12974-018-1332-0 |
| *H2-d1* | CCTTGGAGCTATGGCCATCA | TGTGTTTCTCCTTCTCTTCATCACA | doi: 10.1002/glia.23299 |
| *H2-t23* | GGA CCG CGA ATG ACA TAG C | GCA CCT CAG GGT GAC TTC AT | doi:https://doi.org/10.1186/s40478-019-0767-6 |
| *Iigqp1* | GGG GCA ATA GCT CAT TGG TA | ACC TCG AAG ACA TCC CCT TT | doi:https://doi.org/10.1186/s40478-019-0767-6 |
| *Il-1α* | CGCTTGAGTCGGCAAAGAAAT | CTTCCCGTTGCTTGACGTTG | doi:https://doi.org/10.1186/s40478-019-0767-6 |
| *Il-6* | AACGATGATGCACTTGCAGA | TGGTACTCCAGAAGACCAGAGG | doi: 10.1016/j.ejphar.2014.08.034 |
| *Ifn-β* | AGCTCCAAGAAAGGACGAACAT | GCCCTGTAGGTGAGGTTGATCT | doi: 10.1016/j.ejphar.2014.08.034 |
| *Megf10* | TACCGCCATGGGGAGAAAAC | TTATCAGCGCAGTGAGGGAC | doi: 10.1038/nature21029. |
| *Prseneillin1* | 5'-ACAATGGTGTGGTTGGTGAAT-3 | 5'-AGCAGGCTATGGTTGTGTTCC-3' | doi:10.1111/j.1476-5381.2009.00610.x |
| *Psmb8* | CAGTCCTGAAGAGGCCTACG | CACTTTCACCCAACCGTCTT | doi:https://doi.org/10.1186/s40478-019-0767-6 |
| *Tlr3* | TCATTTTCGTTATCACACACCATTT | TGAACTGCGTGATGTACCTTGAA | doi: 10.1016/j.ejphar.2014.08.034 |
| *Tnf-α* | GCCAGCCGATGGGTTGT | GCAGCCTTGTCCCTTGAAGA | doi: 10.1016/j.ejphar.2014.08.034 |
| *Trem2* | CTGCTGATCACAGCCCTGTCCCAA | CCCCCAGTGCTTCAAGGCGTCATA | [doi: 10.7554/eLife.85279](https://doi.org/10.7554%2FeLife.85279) |

**Supplemental table 2. Antibody information**

| **1st antibody** | **Name** | **Host** | **Company** | **Dilutions** |
| --- | --- | --- | --- | --- |
| AT8 | Phospho-Tau (Ser202, Thr205) Monoclonal Antibody (AT8) | mouse | Invitrogen | 1:100 |
| Iba1 | AIF-1/Iba1 Antibody | goat | Novus Biologicals | 1:250 |
| Iba1 | Iba1 Antibody | rabbit | Wako | 1:500 |
| GFAP | Anti-GFAP Antibody (ab4674) | chicken | Abcam | 1:1000 |
| TAU-5 | Anti-Tau Monoclonal Antibody | mouse | Invitrogen | 1:500 |
| Apoe | Anti-Apoe Antibody | mouse | Abcam | 1:200 |
| Cd11c | Anti-CD11c Antibody | hamster | BD Biosciences | 1:10 |
| NeuN | Anti-NeuN Antibody | mouse | EMD | 1:100 |
| Ser404 | Anti-phospho-Tau (Ser404) | rabbit | Cell signaling | 1:1000 |
| Ser214 | Anti-phospho-Tau (Ser214) | rabbit | Cell signaling | 1:1000 |
| PSD95 | Anti-PSD95 | rabbit | Cell signaling | 1:1000 |
| Synapsin | Anti-Synapsin I | rabbit | Millipore | 1:1000 |
| **2nd antibody** | **Name** | **Host** | **Company** | **Dilutions** |
| Donkey anti-mouse 405 | Donkey anti-mouse IgG (H+L) Alexa Fluor™ 405 | donkey | Invitrogen | 1:1000 |
| Donkey anti-goat 546 | Donkey anti-Goat IgG (H+L) Alexa Fluor™ 546 | donkey | Invitrogen | 1:1000 |
| Donkey anti-chicken 488 | Alexa Fluor® 488 AffiniPure Donkey Anti-Chicken IgY (IgG) (H+L) | donkey | Jackson ImmunoResearch | 1:1000 |
| Donkey anti-goat 488 | Donkey anti-Goat IgG (H+L) Alexa Fluor™ 488 | donkey | Invitrogen | 1:1000 |
| Donkey anti-mouse 594 | Donkey anti-Mouse IgG (H+L) Alexa Fluor™ 594 | donkey | Invitrogen | 1:1000 |
| Donkey anti-mouse 488 | Donkey anti-Mouse IgG (H+L) Alexa Fluor™ 488 | donkey | Invitrogen | 1:1000 |
| Goat anti-mouse 488 | Goat anti-Rabbit IgG (H+L) Alexa Fluor™ 488 | goat | Invitrogen | 1:1000 |
| Goat anti-Hamster 594 | Goat anti-Hamster DyLight594 | goat | BioLegend | 1:500 |

**Supplemental table 3. Regression analysis**

| Behavior | name | Astro *Aqp4* | Astro *Axl* | Astro *Bace1* | Astro *Megf10* | Astro *Psen1* | Micro *Axl* | Micro *Cd11c* | Micro *Cd68* | Micro *Fstl1* | Micro *Il6* | Micro *Tlr3* |
| --- | --- | --- | --- | --- | --- | --- | --- | --- | --- | --- | --- | --- |
| Nest score | estimate | -6.13 | -8.33 | 6.11 | 5.94** | 9.14* | -0.29*** | -0.11*** | -1.69** | 3.11 | 1.63 | 4.48** |
|  | p.value | 0.247 | 0.495 | 0.121 | 0.003 | 0.028 | <0.001 | <0.001 | 0.001 | 0.250 | 0.608 | 0.005 |
|  | conf.int | [-17.43, 5.17] | [-35.19, 18.53] | [-2.01, 14.24] | [2.59, 9.28] | [1.28, 17.00] | [-0.41, -0.16] | [-0.16, -0.06] | [-2.48, -0.89] | [-2.68, 8.91] | [-5.41, 8.66] | [1.80, 7.16] |
|  | Adjust R2 | 0.059 | -0.057 | 0.182 | 0.636 | 0.407 | 0.758 | 0.736 | 0.72 | 0.056 | -0.086 | 0.607 |
| Alteraction | estimate | -35.22 | 12.99 | 41.23 | 21.95 | 17.47 | -0.9 | -0.39 | -6.63 | 46.99* | 6.32 | 16.79 |
|  | p.value | 0.473 | 0.908 | 0.264 | 0.341 | 0.688 | 0.385 | 0.344 | 0.282 | 0.034 | 0.827 | 0.344 |
|  | conf.int | [-142.96, 72.53] | [-236.84, 262.82] | [-38.00, 120.46] | [-28.05, 71.94] | [-79.23, 114.18] | [-3.17, 1.36] | [-1.28, 0.50] | [-19.89, 6.63] | [4.63, 89.35] | [-58.10, 70.75] | [-21.72, 55.31] |
|  | Adjust R2 | -0.05 | -0.123 | 0.047 | 0.003 | -0.101 | -0.018 | 0.001 | 0.035 | 0.381 | -0.118 | 0.001 |
| In open arms(time) | estimate | 236.46 | 289.35 | -312.1 | -273.76*** | -406.39* | 11.51** | 4.65** | 70.22** | -9.35 | -172.49 | -198.98** |
|  | p.value | 0.308 | 0.587 | 0.059 | <0.001 | 0.023 | 0.004 | 0.003 | 0.003 | 0.939 | 0.189 | 0.003 |
|  | conf.int | [-263.98, 736.89] | [-889.87, 1468.57] | [-638.57, 14.37] | [-397.89, -149.63] | [-739.89, -72.89] | [4.94, 18.08] | [2.12, 7.18] | [31.95, 108.49] | [-283.92, 265.21] | [-449.68, 104.70] | [-310.00, -87.96] |
|  | Adjust R2 | 0.02 | -0.082 | 0.3 | 0.734 | 0.434 | 0.63 | 0.653 | 0.653 | -0.124 | 0.105 | 0.641 |
| In closed arms(time) | estimate | -168.56 | -220.52 | 221.05* | 199.03*** | 279.34* | -8.76*** | -3.49*** | -53.07*** | 49.76 | 87.08 | 143.77*** |
|  | p.value | 0.278 | 0.538 | 0.043 | <0.001 | 0.019 | <0.001 | <0.001 | <0.001 | 0.543 | 0.338 | <0.001 |
|  | conf.int | [-502.49, 165.37] | [-1010.06, 569.02] | [8.45, 433.65] | [142.10, 255.96] | [59.50, 499.19] | [-11.67, -5.86] | [-4.64, -2.34] | [-69.75, -36.38] | [-130.68, 230.21] | [-109.80, 283.96] | [82.27, 205.27] |
|  | Adjust R2 | 0.038 | -0.07 | 0.345 | 0.877 | 0.457 | 0.84 | 0.843 | 0.854 | -0.071 | 0.004 | 0.757 |
| sPAL accuracy | estimate | -47.95 | -154.92 | 41.46 | 54.21 | 40.96 | -2.54* | -0.97* | -15.52* | 57.62* | 8.28 | 33.7 |
|  | p.value | 0.430 | 0.231 | 0.379 | 0.072 | 0.453 | 0.038 | 0.046 | 0.031 | 0.022 | 0.806 | 0.108 |
|  | conf.int | [-186.50, 90.60] | [-439.30, 129.46] | [-65.27, 148.20] | [-6.57, 115.00] | [-84.09, 166.02] | [-4.89, -0.19] | [-1.91, -0.03] | [-29.06, -1.98] | [11.57, 103.67] | [-70.70, 87.26] | [-9.98, 77.37] |
|  | Adjust R2 | -0.042 | 0.1 | -0.014 | 0.35 | -0.054 | 0.462 | 0.432 | 0.495 | 0.545 | -0.154 | 0.268 |

**Supplemental table4.****Statistical data of qPCR**

| **Astrocyte** | Number of samples | P/t value |
| --- | --- | --- |
| Tau Astro *Aqp4* (6M) | Control = 5 (Female n = 1 Male n = 4) | P=0.0700 |
|  | rTg4510 = 5 (Female n = 2 Male n = 3) | t=2.09 |
| Tau Astro *Axl* (6M) | Control = 5 (Female n = 1 Male n = 4) | P=0.4349 |
|  | rTg4510 = 5 (Female n = 2 Male n = 3) | t=0.822 |
| Tau Astro *Bace1* (6M) | Control = 5 (Female n = 1 Male n = 4) | P=0.0645 |
|  | rTg4510 = 5 (Female n = 2 Male n = 3) | t=2.143 |
| Tau Astro *Fstl1* (6M) | Control = 5 (Female n = 1 Male n = 4) | P=0.3491 |
|  | rTg4510 = 5 (Female n = 2 Male n = 3) | t=0.9944 |
| Tau Astro *H2-d1* (6M) | Control = 5 (Female n = 1 Male n = 4) | P<0.0001 |
|  | rTg4510 = 5 (Female n = 2 Male n = 3) | t=12.45 |
| Tau Astro *H2-t23* (6M) | Control = 5 (Female n = 1 Male n = 4) | P<0.0001 |
|  | rTg4510 = 5 (Female n = 2 Male n = 3) | t=11.4 |
| Tau Astro *Il6* (6M) | Control = 5 (Female n = 1 Male n = 4) | P=0.0003 |
|  | rTg4510 = 5 (Female n = 2 Male n = 3) | t=6.045 |
| Tau Astro *Ligp1* (6M) | Control = 5 (Female n = 1 Male n = 4) | P=0.0034 |
|  | rTg4510 = 5 (Female n = 2 Male n = 3) | t=4.117 |
| Tau Astro *Megf10* (6M) | Control = 5 (Female n = 1 Male n = 4) | P<0.0001 |
|  | rTg4510 = 5 (Female n = 2 Male n = 3) | t=9.497 |
| Tau Astro *Psen1* (6M) | Control = 5 (Female n = 1 Male n = 4) | P=0.0072 |
|  | rTg4510 = 5 (Female n = 2 Male n = 3) | t=3.575 |
| Tau Astro *Psmb8* (6M) | Control = 5 (Female n = 1 Male n = 4) | P<0.0001 |
|  | rTg4510 = 5 (Female n = 2 Male n = 3) | t=12.09 |

| **Microglia** | Number of samples | P/t value |
| --- | --- | --- |
| Tau Micro *Apoe* (6M) | Control = 5 (Female n = 1 Male n = 4) | P<0.0001 |
|  | rTg4510 = 5 (Female n = 2 Male n = 3) | t=37.59 |
| Tau Micro *Axl* (6M) | Control = 5 (Female n = 1 Male n = 4) | P<0.0001 |
|  | rTg4510 = 5 (Female n = 2 Male n = 3) | t=24.23 |
| Tau Micro *C1q* (6M) | Control = 5 (Female n = 1 Male n = 4) | P<0.0001 |
|  | rTg4510 = 5 (Female n = 2 Male n = 3) | t=13.38 |
| Tau Micro *Cd11c* (6M) | Control = 5 (Female n = 1 Male n = 4) | P<0.0001 |
|  | rTg4510 = 5 (Female n = 2 Male n = 3) | t=54.35 |
| Tau Micro *Cd68* (6M) | Control = 5 (Female n = 1 Male n = 4) | P<0.0001 |
|  | rTg4510 = 5 (Female n = 2 Male n = 3) | t=35.62 |
| Tau Micro *Cst7* (6M) | Control = 5 (Female n = 1 Male n = 4) | P<0.0001 |
|  | rTg4510 = 5 (Female n = 2 Male n = 3) | t=13.18 |
| Tau Micro *Fstl1* (6M) | Control = 5 (Female n = 1 Male n = 4) | P=0.1580 |
|  | rTg4510 = 5 (Female n = 2 Male n = 3) | t=1.557 |
| Tau Micro *Ifn-β* (6M) | Control = 5 (Female n = 1 Male n = 4) | P=0.0004 |
|  | rTg4510 = 5 (Female n = 2 Male n = 3) | t=5.746 |
| Tau Micro *Il-1α* (6M) | Control = 5 (Female n = 1 Male n = 4) | P=0.0087 |
|  | rTg4510 = 5 (Female n = 2 Male n = 3) | t=3.447 |
| Tau Micro *Il6* (6M) | Control = 5 (Female n = 1 Male n = 4) | P=0.1210 |
|  | rTg4510 = 5 (Female n = 2 Male n = 3) | t=1.735 |
| Tau Micro *Tlr3* (6M) | Control = 5 (Female n = 1 Male n = 4) | P<0.0001 |
|  | rTg4510 = 5 (Female n = 2 Male n = 3) | t=17.32 |
| Tau Micro *Tnf-α* (6M) | Control = 5 (Female n = 1 Male n = 4) | P<0.0001 |
|  | rTg4510 = 5 (Female n = 2 Male n = 3) | t=14.33 |
| Tau Micro *Trem2* (6M) | Control = 5 (Female n = 1 Male n = 4) | P=0.0028 |
|  | rTg4510 = 5 (Female n = 2 Male n = 3) | t=4.241 |

**Supplemental table 5.Statistical data of Behavior and immunostaining**

| **Figure** | **Number of samples** | **Test used** | **Degree of freedom and F/t/P value** | **Post-hoc test** | **Significance** |
| --- | --- | --- | --- | --- | --- |
| **Figure1.B** | Control = 9 (Female n = 5 Male n = 4) | Unpaired t test | P<0.0001 |  |  |
|  | rTg4510 = 9 (Female n = 5 Male n = 4) |  | t=6.261 |  |  |
| **Figure1.C** | Control = 9 (Female n = 5 Male n = 4) | Unpaired t test | P=0.0741 |  |  |
|  | rTg4510 = 9 (Female n = 5 Male n = 4) |  | t=1.911 |  |  |
| **Figure1.D** | Control = 9 (Female n = 5 Male n = 4) | Unpaired t test | P<0.0001 |  |  |
|  | rTg4510 = 9 (Female n = 5 Male n = 4) |  | t=5.268 |  |  |
| **Figure1.E** | Control = 9 (Female n = 5 Male n = 4) | Unpaired t test | P<0.0001 |  |  |
|  | rTg4510 = 9 (Female n = 5 Male n = 4) |  | t=8.962 |  |  |
| **Figure1.F** | Control = 9 (Female n = 5 Male n = 4) | Unpaired t test | P=0.0618 |  |  |
|  | rTg4510 = 9 (Female n = 5 Male n = 4) |  | t=2.008 |  |  |
| **Figure2.A** | Control = 10 (Female n = 5 Male n = 5) | Unpaired t test | P<0.0001 |  |  |
|  | rTg4510 = 9 (Female n = 5 Male n = 4) |  | t=12.06 |  |  |
| **Figure2.B** | Control = 10 (Female n = 5 Male n = 5) | Unpaired t test | P=0.0404 |  |  |
|  | rTg4510 = 9 (Female n = 5 Male n = 4) |  | t=2.218 |  |  |
| **Figure2.C** | Control = 10 (Female n = 5 Male n = 5) | Unpaired t test | P<0.0001 |  |  |
|  | rTg4510 = 8 (Female n = 4 Male n = 4) |  | t=7.711 |  |  |
| **Figure2.D** | Control = 10 (Female n = 5 Male n = 5) | Unpaired t test | P<0.0001 |  |  |
|  | rTg4510 = 8 (Female n = 4 Male n = 4) |  | t=7.571 |  |  |
| **Figure2.E** | Control = 10 (Female n = 5 Male n = 5) | Unpaired t test | P=0.0286 |  |  |
|  | rTg4510 = 8 (Female n = 4 Male n = 4) |  | t=2.406 |  |  |

| **Figure** | **Number of samples** | **Test used** | **Degree of freedom and F/t/P value** | **Post-hoc test** | **Significance** |
| --- | --- | --- | --- | --- | --- |
| **Figure3.B** | Control = 9 (Female n = 3 Male n = 6) | Two-way ANOVA | Gene : F (1, 15) = 14.81 , P=0.0016 | Sidak's multiple comparisons test | Day9 : P=0.0195 |
|  | rTg4510 = 8 (Female n = 4 Male n = 4) |  | Time : F (13, 195) = 14.02 , P<0.0001 |  | Day10 : P=0.0015 |
|  |  |  | Gene×Time : F (13, 195) = 2.974 , P=0.0005 |  | Day12 : P=0.0007 |
|  |  |  |  |  | Day13 : P<0.0001 |
|  |  |  |  |  | Day14 : P=0.0002 |
| **Figure3.D** | Control=13 (Female n = 6 Male n = 7) | Two-way ANOVA | Gene : F (1, 23) = 33.16 , P<0.0001 | Sidak's multiple comparisons test | Day2 : P=0.0028 |
|  | rTg4510=12 (Female n = 7 Male n = 5) |  | Time : F (3, 69) = 47.95 , P<0.0001 |  | Day3 : P<0.0001 |
|  |  |  | Gene×Time : F (3, 69) = 4.477 , P=0.0062 |  | Day4 : P=0.0002 |
| **Figure3.E** | Control=13 (Female n = 6 Male n = 7) | Unpaired t test | Control vs. rTg4510 :P=0.0086 |  |  |
|  | rTg4510=12 (Female n = 7 Male n = 5) |  |  |  |  |
| **Figure3.F** | Control=12 (Female n = 5 Male n = 7) | Two-way ANOVA | Gene : F (1, 18) = 0.8716 , P=0.3629 | Sidak's multiple comparisons test |  |
|  | rTg4510=8 (Female n = 3 Male n = 5) |  | Time : F (5, 90) = 84.50 , P<0.0001 |  |  |
|  |  |  | Gene×Time : F (5, 90) = 4.614 , P=0.0009 |  |  |
| **Figure4.C** | Control = 3 (Female n = 1 Male n = 2) | Unpaired t test | P=0.0050 |  |  |
|  | rTg4510 = 3 (Female n = 1 Male n = 2) |  | t=3.251 |  |  |
| **Figure4.F** | Control = 3 (Female n = 1 Male n = 2) | Unpaired t test | P=0.0002 |  |  |
|  | rTg4510 = 3 (Female n = 1 Male n = 2) |  | t=4.829 |  |  |
| **Figure4.I** | Control = 3 (Female n = 1 Male n = 2) | Unpaired t test | P<0.0001 |  |  |
|  | rTg4510 = 3 (Female n = 1 Male n = 2) |  | t=5.582 |  |  |
| **Figure4.J** | Control = 3 (Female n = 1 Male n = 2) | Unpaired t test | P=0.0001 |  |  |
|  | rTg4510 = 3 (Female n = 1 Male n = 2) |  | t=4.947 |  |  |

| **Figure** | **Number of samples** | **Test used** | **Degree of freedom and F/t/P value** | **Post-hoc test** | **Significance** |
| --- | --- | --- | --- | --- | --- |
| **Supplemental figure1.B** | 3m Control =4 (Female n = 3 Male n = 1) | One-way ANOVA | 3m control vs 3m rTg4510 P < 0.01 |  |  |
|  |  |  | 6m control vs 6m rTg4510 P < 0.05 |  |  |
| **Supplemental figure1.C** |  | One-way ANOVA | 3m control vs 3m rTg4510 P<0.001 |  |  |
|  | 3m rTg4510 =4 (Female n = 1 Male n = 3) |  | 6m control vs 6m rTg4510 P < 0.05 |  |  |
| **Supplemental figure1.D** |  | One-way ANOVA | 3m control vs 3m rTg4510 P < 0.001 |  |  |
|  |  |  | 6m control vs 6m rTg4510 P < 0.05 |  |  |
| **Supplemental figure1.E** | 6m Control =4 (Female n = 4 Male n = 0) | One-way ANOVA | 3m control vs 3m rTg4510 P<0.001 |  |  |
|  |  |  | 6m control vs 6m rTg4510 P<0.05 |  |  |
| **Supplemental figure1.F** |  | One-way ANOVA | 3m control vs 3m rTg4510 P<0.01 |  |  |
|  | 6m rTg4510 =4 (Female n = 4 Male n = 0) |  | 6m control vs 6m rTg4510 P < 0.05 |  |  |
| **Supplemental figure1.G** |  | One-way ANOVA | 3m control vs 3m rTg4510 P<0.001 |  |  |
|  |  |  | 6m control vs 6m rTg4510 P<0.05 |  |  |

| **Figure** | **Number of samples** | **Test used** | **Degree of freedom and F/t/P value** | **Post-hoc test** | **Significance** |
| --- | --- | --- | --- | --- | --- |
| **Supplemental figure2.A** | Control =9 (Female n = 5 Male n = 4) | Two-way ANOVA | Interaction : F (23, 368) = 0.7588 , P=0.7823 | Sidak's multiple comparisons test |  |
|  | rTg4510 =9 (Female n = 5 Male n = 4) |  | Time : F (23, 368) = 10.44 , P<0.0001 |  |  |
|  |  |  | Gene : F (1, 16) = 0.8555 , P=0.3687 |  |  |
|  |  |  | Subjects (matching) : F (16, 368) = 6.511 , P<0.0001 |  |  |
| **Supplemental figure2.B** | Control =9 (Female n = 5 Male n = 4) | Unpaired t test | P=0.2482 |  |  |
|  | rTg4510 =9 (Female n = 5 Male n = 4) |  | t=1.198 |  |  |
| **Supplemental figure3.A** | Control = 8 (Female n = 3 Male n = 5) | Two-way ANOVA | Interaction : F (23, 322) = 0.9391 , P=0.5457 | Sidak's multiple comparisons test |  |
|  | rTg4510 = 8 (Female n = 5 Male n = 3) |  | Time : F (23, 322) = 7.893 , P<0.0001 |  |  |
|  |  |  | Gene : F (1, 14) = 0.6930 , P=0.4191 |  |  |
|  |  |  | Subjects (matching) : F (14, 322) = 1.175 , P=0.2933 |  |  |
| **Supplemental figure3.B** | Control = 8 (Female n = 3 Male n = 5) | Unpaired t test | P<0.0001 |  |  |
|  | rTg4510 = 8 (Female n = 5 Male n = 3) |  | t=6.700 |  |  |
| **Supplemental figure3.C** | Control =9 (Female n = 5 Male n = 4) | Unpaired t test | P=0.1258 |  |  |
|  | rTg4510 =9 (Female n = 5 Male n = 4) |  | t=1.615 |  |  |
| **Supplemental figure3.D** | Control =9 (Female n = 5 Male n = 4) | Unpaired t test | P=0.0105 |  |  |
|  | rTg4510 =9 (Female n = 5 Male n = 4) |  | t=2.897 |  |  |
| **Supplemental figure4.B** | Control = 3 (Female n = 1 Male n = 2) | Unpaired t test | P=0.0063 |  |  |
|  | rTg4510 = 3 (Female n = 1 Male n = 2) |  | t=3.143 |  |  |
| **Supplemental figure4.D** | 6m Control =4 (Female n = 4 Male n = 0) | Unpaired t test | P=0.2928 |  |  |
|  | 6m rTg4510 =4 (Female n = 4 Male n = 0) |  | t=1.153 |  |  |
| **Supplemental figure4.E** | 6m Control =4 (Female n = 4 Male n = 0) | Unpaired t test | P=0.6044 |  |  |
|  | 6m rTg4510 =4 (Female n = 4 Male n = 0) |  | t=0.5465 |  |  |
